# ACO2 deficiency increases vulnerability to Parkinson’s disease via dysregulating mitochondrial function and histone acetylation-mediated transcription of autophagy genes

**DOI:** 10.1038/s42003-023-05570-y

**Published:** 2023-11-25

**Authors:** Junge Zhu, Fanxi Xu, Hong Lai, Huiyao Yuan, Xu-Ying Li, Junya Hu, Wei Li, Lei Liu, Chaodong Wang

**Affiliations:** 1 Department of Neurology & Neurobiology, Xuanwu Hospital of Capital Medical University, National Clinical Research Center for Geriatric Diseases, Beijing, 100053 China; 2 Department of Neurology, The First Affiliated Hospital of Gannan Medical University, Ganzhou, 341000 China; 3 Department of Biochemistry and Molecular Biology, Capital Medical University; School of Basic Medicine, Beijing, 100069 China; 4 Department of Stroke Center, Central Hospital Affiliated to Shandong First Medical University, Jinan, 250000 China

**Keywords:** Parkinson's disease, Cell death in the nervous system

## Abstract

Parkinson’s disease (PD) is characterized by α-synuclein aggregation in dopaminergic (DA) neurons, which are sensitive to oxidative stress. Mitochondria aconitase 2 (ACO2) is an essential enzyme in the tricarboxylic acid cycle that orchestrates mitochondrial and autophagic functions to energy metabolism. Though widely linked to diseases, its relation to PD has not been fully clarified. Here we revealed that the peripheral ACO2 activity was significantly decreased in PD patients and associated with their onset age and disease durations. The knock-in mouse and *Drosophila* models with the A252T variant displayed aggravated motor deficits and DA neuron degeneration after 6-OHDA and rotenone-induction, and the ACO2 knockdown or blockade cells showed features of mitochondrial and autophagic dysfunction. Moreover, the transcription of autophagy-related genes *LC3* and *Atg5* was significantly downregulated via inhibited histone acetylation at the H3K9 and H4K5 sites. These data provided multi-dimensional evidences supporting the essential roles of ACO2, and as a potential early biomarker to be used in clinical trials for assessing the effects of antioxidants in PD. Moreover, ameliorating energy metabolism by targeting ACO2 could be considered as a potential therapeutic strategy for PD and other neurodegenerative disorders.

## Abstract (web summary)

ACO2 activity is associated with the age of onset for Parkinson’s disease (PD), and the PD-related ACO2 A252T variant causes mitochondrial and autophagy dysfunction, leading to behavioral deficits and dopaminergic neuron loss in PD models.

## Introduction

Parkinson’s disease (PD) is a common neurodegenerative disorder that affect approximately 9 million people worldwide^1^. The clinical features of the disease consist of motor symptoms including bradykinesia, rest tremor and rigidity, and a wide range of non-motor symptoms^2,3^. Pathologically, it is mainly characterized by progressive loss of dopaminergic (DA) neurons in the substantia nigra (SN) and the formation of Lewy bodies (LBs) in neurons^4–6^. In most populations, 3–5% of PD is familial and caused by monogenic mutations, while 16–36% of genetic risk for sporadic cases were explained by about 90 genetic risk variants^7^. The mutant proteins of these genes lead to the degeneration of DA neurons via dysfunctions of mitochondria and the autophagic lysosomal protein degradation pathway^8–10^.

There are increasing evidences supporting a direct association between the incidence and progression of neurodegenerative disorders and impaired energy metabolism in neuronal cells^11,12^. In mitochondria, the tricarboxylic acid (TCA) cycle plays a role in both energy production and biosynthesis. Aconitase 2 (ACO2) is a member of the iron-sulfur cluster [4Fe-4S] hydrase family, and the active [4Fe-4S] cluster, in combination with other structures, reversibly catalyzes citric acid to isocitrate in the TCA cycle and promotes dehydration and rehydration reactions^13,14^. ACO2 plays an important role in cellular energy metabolism, the maintenance of iron homeostasis, oxidative stress defense and the integrity of mitochondrial DNA (mtDNA)^15,16^. Most recently, recessive or dominant ACO2 variants have been extensively reported in cases displaying problems of energy metabolism and progression of neurodegenerative disorders, including optic nerve atrophy, microcephaly, intellectual disability, cognitive decline, hypotonia and spastic paraplegia, as well as neuromuscular condition^17–21^. Moreover, it has been proposed as a biomarker of multiple types of cancers^22,23^.

The enzyme activity of ACO2 is regulated by a variety of factors, mainly including the attack of [4Fe-4S] cluster by reactive oxygen species^24^, post-translational modification of ACO2^25^, and *ACO2* variation^19^. Previous studies have shown that ACO2 activity was decreased in cellular and animal models of PD. Rotenone and 6-OHDA significantly reduced the ACO2 activity^26,27^. In addition, the activity of ACO2 was markedly decreased in the brain of DJ-1-knockout mice, which may be related to the excessive oxygen free radicals^28^. Moreover, in *Drosophila* PD model with PINK1 mutation, oxidative inactivation of ACO2 can induce iron-mediated oxidative stress, resulting in mitochondrial swelling and increased permeability^29^. Similarly, ACO2 activity was significantly reduced in striatum (STR) of PINK1-knockout mice^30^. These studies demonstrated that the vicious cycle between the environmental toxins and oxygen free radicals produced by the [4Fe-4S] cluster of ACO2 will further lead to the degeneration of DA neurons. However, definite evidence on the roles of ACO2, especially the detailed molecular mechanisms in the vulnerability to PD is lacking.

To address the questions, we measured the ACO2 activity in PBMCs and screened for rare coding variants of *ACO2* in PD patients. We determined the molecular mechanisms underlying PD using the *Aco2* knock-in (KI) mouse and *Drosophila* models using the activity-reducing variant, as well as cell models with *Aco2* RNAi-based knockdown or specific blockade by tricarballylic acid (TA). We revealed that the motor deficits related to the ACO2 deficiency are caused by the ablation of ACO2-dependent regulations of mitochondrial and nuclear epigenetic modulations for autophagy genes, which can be partially rescued by CoQ10.

## Results

### Decreased ACO2 activity in peripheral blood mononuclear cells (PBMCs) of PD patients and its association with PD clinical features

**Fig. 1:**
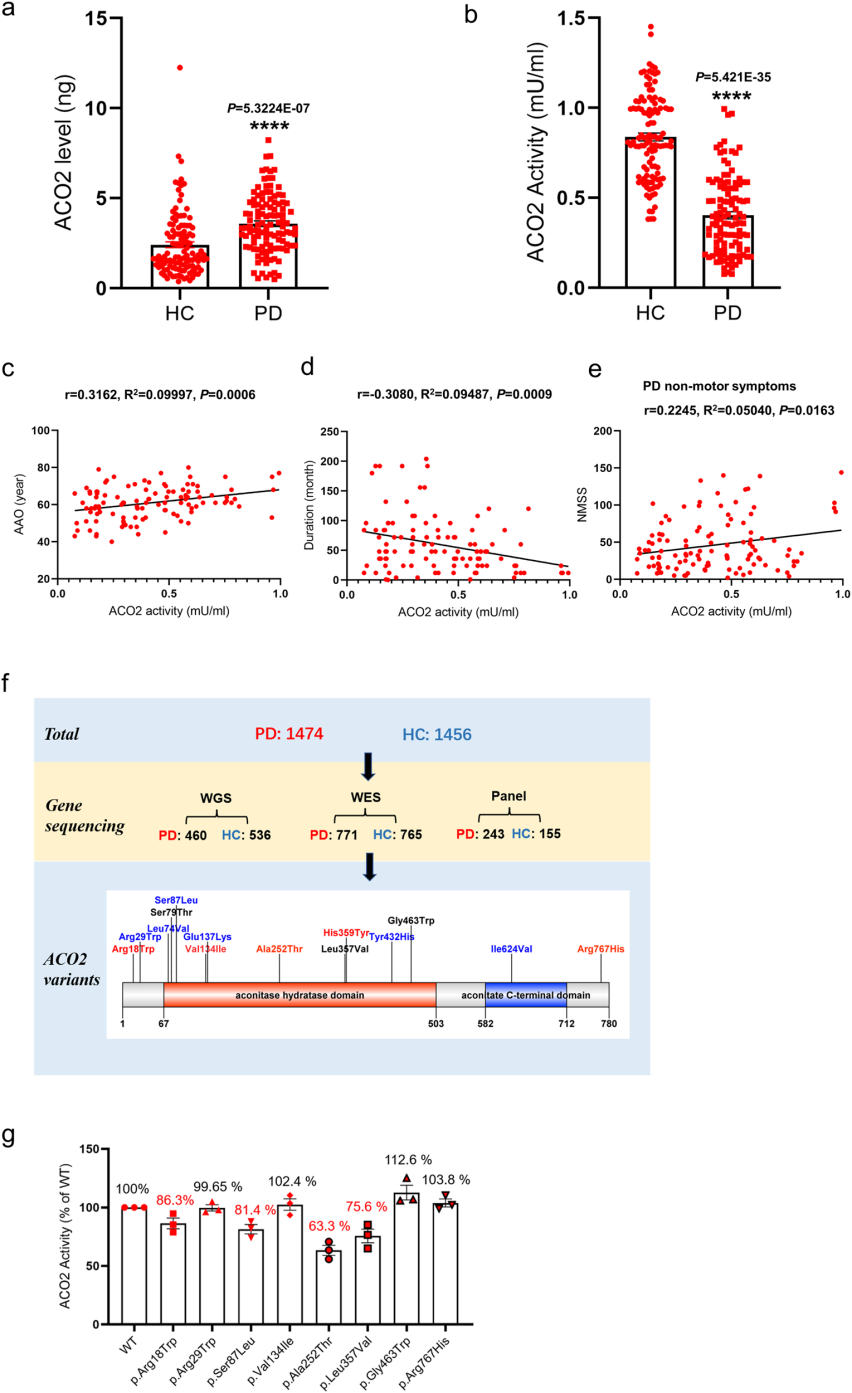
ACO2 protein expression levels and ACO2 activity in PBMC of HC and PD patients, and genetic analysis and the activity of human *ACO2* variants. **a** Elisa assay for ACO2 expression in PBMC of PD and HC. **b** ACO2 activity in PBMC of PD and HC. *n* = 114 and 117 for PD and HC groups, respectively. ****P < 0.0001 vs HC. Differences among means were determined by unpaired *t* test. **c** Correlations between ACO2 activity and AAO of PD. **d** Correlations between ACO2 activity and duration of PD. **e** Correlations between ACO2 activity and NMSS of PD. **f** Schematic representation of the location of rare variants in ACO2 protein. The variation found in PD and HC are mapped to the ACO2 protein, red represents the variation carried by PD, blue represents the variation carried by HC, and black represents the variation carried by both PD and HC. **g** Activity of WT and variant human ACO2 protein purified from E.coli. *n* = 3. Error bars indicate mean ± SEM. All the *P* values were two-sided. Source data are available as a Supplementary Data 1 file.

To evaluate the changes of ACO2 in PD patients, we assayed its expression and enzymatic activity in PBMCs from 114 PD patients (age: 66.02 ± 0.73; gender: male/female = 56/58) and 117 HCs (age: 64.12 ± 0.47; gender: male/female = 51/66). We found that the expression of the ACO2 protein in PBMCs was significantly higher in PD patients than in HCs (Fig. 1a), which were even higher in patients with well-water drinking and pesticide exposure (*P* = 0.0009 and *P* = 0.0003, respectively) (Table 1). In contrast, the ACO2 activity was significantly lower in PD patients than in HCs (Fig. 1b). In addition, the activity was further reduced in patients with well-water drinking and exposure to pesticides (*P* = 0.0003 and *P* = 0.0008, respectively) (Table 1). However, gender, age, Hoehn & Yahr scale and tea drinking of PD patients were not significantly associated with the ACO2 activity (Table 1). Moreover, linear regression analysis showed that ACO2 activity in PBMCs of PD patients was positively correlated with age at onset (AAO) (R^2^ = 0.09997, *P* = 0.0006) and non-motor symptoms (NMSS) (R^2^ = 0.05040, *P* = 0.0163) (Fig. 1c, e), and was negatively correlated with disease duration (Fig. 1d), but was not significantly associated with H&Y stage and other symptomatic scores (Supplementary Fig. 1). These data suggest that ACO2 might be a sensitive marker for the onset and duration of PD, which reflects life style and exposures to oxidative stress-related environmental toxins.

**Table 1:** The ACO2 expression and enzymatic activity in PBMCs from PD patients.

Clinical or demographic features	Number	ACO2 expression (ng/mg)	*P* value	ACO2 activity (mU/ml)	*P* value
Gender					
Male	56	3.588 ± 0.2206	0.9868	0.3890 ± 0.02804	0.2092
Female	58	3.583 ± 0.2155		0.4416 ± 0.03072	
Age					
<65	43	3.863 ± 0.2287	0.1604	0.3860 ± 0.03073	0.2686
≥65	71	3.417 ± 0.2024		0.4338 ± 0.02784	
Hoehn & Yahr scale					
≤2	77	3.763 ± 0.1823	0.0957	0.4103 ± 0.02606	0.6566
>2	37	3.216 ± 0.2760		0.4302 ± 0.03404	
Well-water drinking					
Yes	66	4.013 ± 0.1885	0.0009	0.3532 ± 0.02251	0.0003
No	48	2.998 ± 0.2328		0.5019 ± 0.03542	
Pesticide exposure					
Yes	33	4.429 ± 0.2443	0.0003	0.3081 ± 0.02835	0.0008
No	81	3.242 ± 0.1789		0.4597 ± 0.02556	
Tea drinking					
Yes	39	3.405 ± 0.2232	0.4002	0.3960 ± 0.03383	0.4970
No	75	3.679 ± 0.2025		0.4261 ± 0.02651	

### Rare ACO2 variants identified in PD patients that lead to decreased ACO2 activity

Multiple mutations and variants have been reported to change the ACO2 enzymatic activity in a series of diseases, including neurodegenerative disorders. Here we went on to screen the existence of similar variants in *ACO2* in PD patients. We collected the sequencing data from our several genetic studies using whole-genome, whole-exome and targeted panel gene sequencing technologies, which totally involved 1474 PD cases and 1456 HCs. As a result, 14 rare coding variants (MAF < 0.01) were identified, among which 34 (2.3%) were carried by PD patients and 18 (1.2%) by HCs (OR = 1.88, *P* = 0.029) (Table 2; Fig. 1f). Moreover, we measured the activities of the protein carrying these variants and found that four variants, Arg18Trp, Ser87Leu, Ala252Thr and Leu357Val, cause activity reduction to varying degrees (Fig. 1g). Of these, the Ala252Thr (A252T) detected in a PD patient most significantly decreased to 63.3% of the WT protein (Fig. 1g, Sanger sequencing of A252T is shown in Supplementary Fig. 2c).

**Table 2:** *ACO2* rare coding variants identified in PD and HC.

Rare variant	Exon	Functional domain	PD (*n* = 1474)	HC (*n* = 1456)
c.52 C > T; p.Arg18Trp	1	-	2	0
c.85 C > T; p.Arg29Trp	2	-	0	1
c.220 C > G; p.Leu74Val	3	Aconitase hydratase domain	0	1
c.236 G > C; p.Ser79Thr	3	Aconitase hydratase domain	2	2
c.260 C > T; p.Ser87Leu	3	Aconitase hydratase domain	0	1
c.400 G > A; p.Val134Ile	3	Aconitase hydratase domain	1	0
c.409 G > A; p.Glu137Lys	3	Aconitase hydratase domain	0	2
c.754 G > A; p.Ala252Thr	6	Aconitase hydratase domain	1	0
c.1069 C > G; p.Leu357Val	9	Aconitase hydratase domain	1	1
c.1075 C > T; p.His359Tyr	9	Aconitase hydratase domain	1	0
c.1294 T > C; p.Tyr432His	10	Aconitase hydratase domain	0	1
c.1387 G > T; p.Gly463Trp	11	Aconitase hydratase domain	25	8
c.1870A>G; p.Ile624Val	15	Aconitase C-terminal domain	0	1
c.2300 G > A; p.Arg767His	18	-	1	0
Total			34	18

### Homozygous A252T variant aggravates the motor deficits associated with loss of DA neurons in a mouse model for PD

**Fig. 2:**
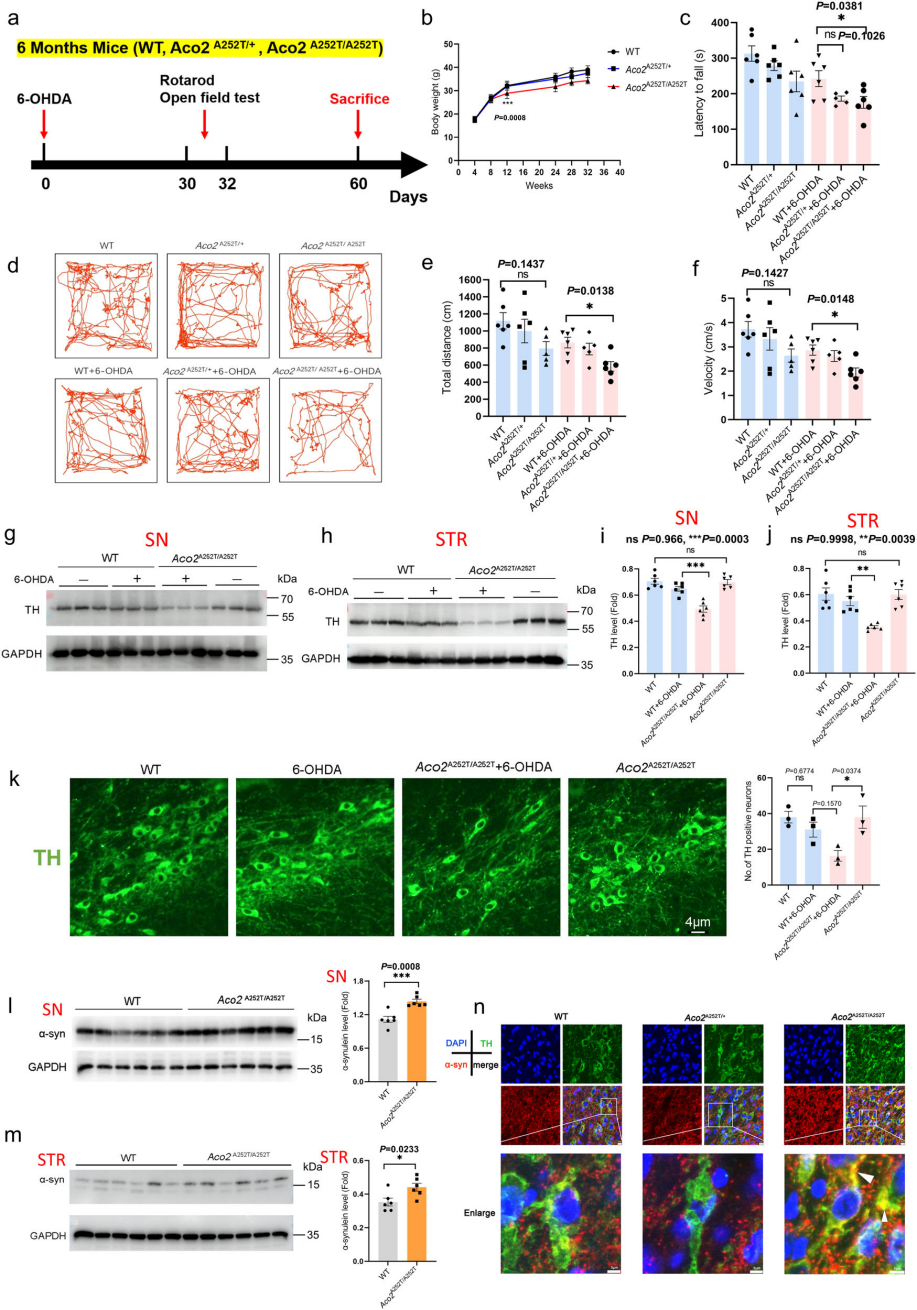
*Aco2*-A252T variation aggravates movement deficits and DA neurons degeneration in 6-OHDA-treated mice. **a** Experimental design. **b** Mice body weight at 4, 8, 12, 24, 28 and 32 weeks. *n* = 12 (4, 8, 12, 24 weeks), *n* = 6 (28 and 32 weeks). Black line: WT mice; blue line: heterozygous mice; red line: homozygous mice. **c** The motor coordination of 7 months mice was tested by rotarod test. *n* = 5–6. **d** Representative traces of mice in Open-field test. **e** Total distances traveled in the open-field (7 months mice). *n* = 5–6. **f** Velocity traveled in the open-field (7 months mice). *n* = 5–6. (**g** and **h**) Expression levels of TH in the SN and STR were determined by Western blotting, respectively. (**i** and **j**) Quantification of TH expression in the SN and STR. *n* = 6. **k** Immunofluorescent staining and quantification of TH^+^ neurons in the SN*pc* (scale bars, 4 µm), *n* = 3 (8 months mice). TH^+^ neurons were stained in green. **l** Expression levels and quantification of α-syn in SN were determined by Western blotting, *n* = 6 (8 months mice). **m** Expression levels and quantification of α-syn in STR were determined by Western blotting, *n* = 6 (8 months mice). **n** Immunofluorescent staining of α-syn (red) in the SN*pc*, TH^+^ neurons were stained in green, and the nuclei were stained with DAPI (blue) (scale bars: 10 µm; enlarged scale bars: 5 µm). *n* = 3 (8 months mice). ***P < 0.001, **P < 0.01 and *P < 0.05. Statistical significance was performed with an unpaired *t* test between two groups, and a one-way ANOVA with Tukey’s post hoc test was used for comparing more than two groups. Error bars indicate mean ± SEM. All the *P* values were two-sided. Source data are available as a Supplementary Data 1 file.

The significantly reduced ACO2 activity in PD cases raises the question whether the protein had regulatory roles in the mechanisms of the disease. Moreover, the identification of the activity-reducing variant in the gene makes it easy to monitor the deficient activity and investigate its role in the PD pathogenesis. We first generated the knock-in (KI) mice for the A252T variant (c.754 G > A, p.Ala252Thr), which was identical to human variant (Supplementary Fig. 2a, e), and passaged and crossed them to produce the homozygous KI mice (*Aco2*^A252T/A252T^ mice) (Supplementary Fig. 2f). We then induced the mice using the 6-OHDA to generate the PD model and performed behavioral tests 30 days later (Fig. 2a). We showed that the body weight (at 12 weeks) of the homozygous KI mice was markedly lower than the WT mice, but no significant difference was observed between the heterozygous KI (*Aco2*^A252T/+^ mice) and WT mice (Fig. 2b). Furthermore, 6 months-old mice received two parallel sites infusion of 2 μl 6-OHDA (7.5 µg, which decreased TH expression, but not significantly, Supplementary Fig. 3) or vehicle solution into the unilateral striatum. After the 6-OHDA treatment for 30 days, both the WT and KI mice showed shorter latency to fall, decreased velocity and distance for traveling in the open field, and these deficits were much more significantly in the KI, especially in the homozygous KI, mice (Fig. 2c–f). Meanwhile, the body weight was significantly reduced in homozygous KI (or 6-OHDA + homozygous KI) compared to the WT (or 6-OHDA + WT) mice (Supplementary Fig. 4). However, the DA neurons were slightly decreased in the 6-OHDA treated homozygous mice, as compared to the WT after the 6-OHDA treatment for 30 days (Supplementary Fig. 5).

After 6-OHDA/saline injection for 60 days, the mice were sacrificed for pathological studies. The expression of tyrosine hydroxylase (TH) in substantia nigra (SN) and striatum (STR) was mildly decreased in WT mice when treated with 6-OHDA, but was significantly reduced in the homozygous KI mice with the induction (Fig. 2g–j), while there was no significant alteration in heterozygous KI mice with the induction (Supplementary Fig. 6a–d). The reduction of the TH-positive neurons in the homozygous KI + 6-OHDA mice was also evidenced in the immunofluorescence staining (Fig. 2k).

We also explored whether the variant causes increased α-synuclein (α-syn) in the SN and STR. As a result, the α-syn expression was significantly increased in the SN and STR of homozygotes KI mice compared with the WT mice (Fig. 2l, m). In addition, it was deposited in the TH-positive DA neurons of SN*pc* region of the homozygous KI, but not the heterozygous KI and WT mice (Fig. 2n). Furthermore, the phospho-α-synuclein (p-α-syn) was increased in the SN DA neurons of homozygous KI mice (Supplementary Fig. 7).

### Decreased activity of ACON aggravated rotenone-induced behavioral deficits and neurodegeneration in flies

**Fig. 3:**
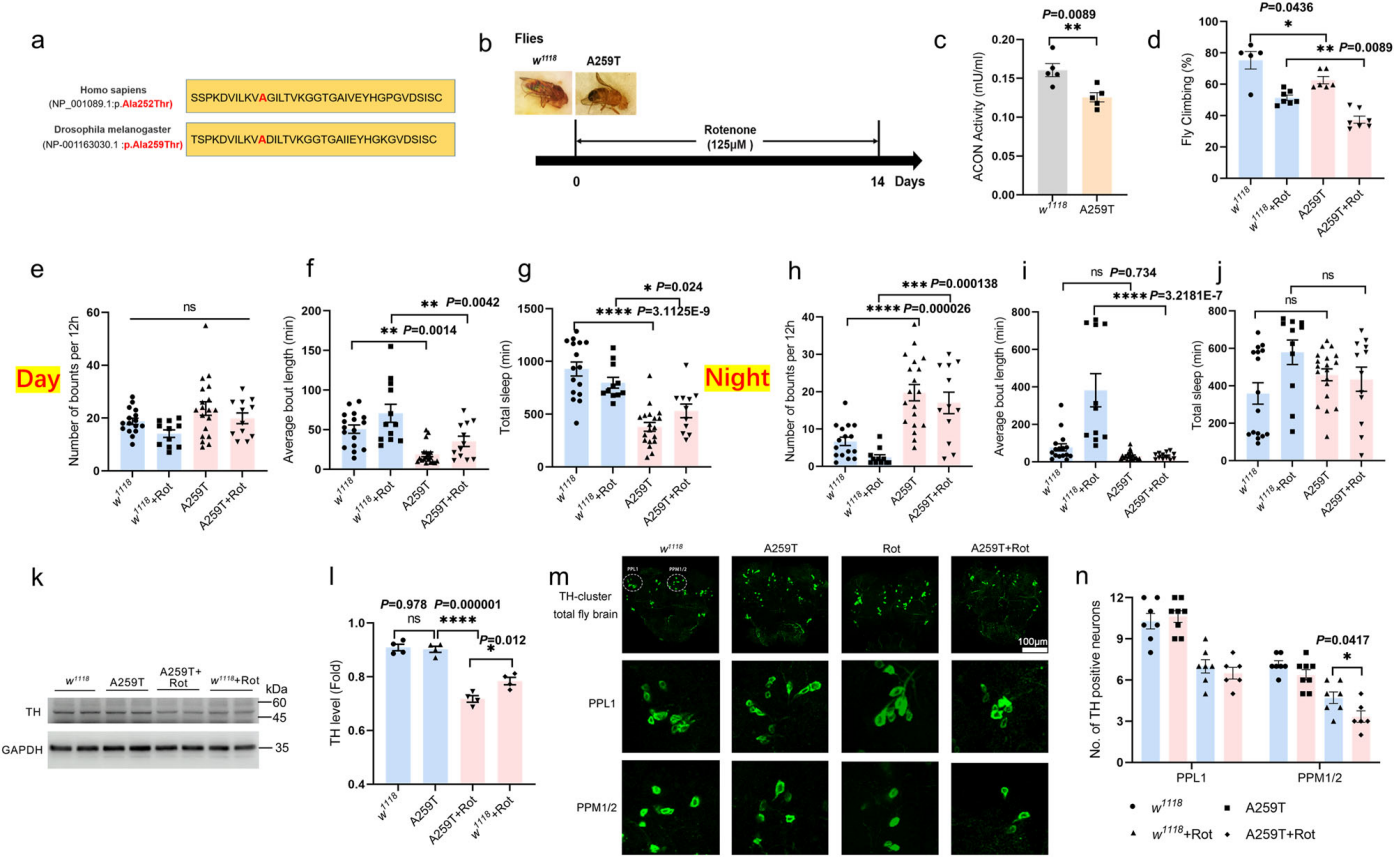
*Acon*-A259T variation aggravates the damage of DA neurons and movement deficits in Rot-treated flies. **a** The high homology of the A252 site (A259 site in the Fly) between human and *Drosophila melanogaster* of ACO2 protein sequence. **b** Experimental design. 36 days *w*^*1118*^ and *Acon*-A259T (A259T) flies received Rot (125 μM in food) for 14 days. Flies were raised and kept under a 12 h light/12 h day cycle. **c** ACON activity. *n* = 4. **d** Climbing behavior of flies. *n* = 5, 7, 6, and 7 for *w*^*1118*^, *w*^*1118*^ +Rot, A259T and A259T+Rot groups, respectively. **e**, **h** Average number of sleep episodes during the light and dark (day and night). **f** and **i** Average length of sleep per episode during the light and dark. **g**, **j** Total length of sleep during the light and dark. Data points represent individual flies, and results are expressed as the mean ± SEM. *n* = 16, 11, 19, and 12 for *w*^*1118*^, *w*^*1118*^ +Rot, A259T and A259T+Rot groups, respectively. **k**, **l** Western blotting and quantitative analysis for TH in fly heads. 100 fly heads were collected for each experiment. *n* = 4. **m** Immunofluorescent staining of TH^+^ neurons in fly heads (scale bars, 100 µm). TH^+^ neurons were stained in green. **n** Quantitative analysis for TH^+^ neurons in PPL1 and PPM1/2 cluster of fly heads. *n* = 7, 8, 7, and 6 for *w*^*1118*^, A259T, *w*^*1118*^ +Rot and A259T+Rot groups, respectively. ****P < 0.0001, ***P < 0.001, **P < 0.01 and *P < 0.05. Statistical significance was performed with an unpaired *t* test between two groups, and a one-way ANOVA with Tukey’s post hoc test was used for comparing more than two groups. All the *P* values were two-sided. Source data are available as a Supplementary Data 1 file.

The *Drosophila* homolog of human ACO2 is ACON. To further validate the ACON effect on motor dysfunction in PD, we also generate the *Drosophila* KI model for the A259T variant. As a conserved protein, the Ala252 amino acid in *hACO2* is equivalent to the Ala259 of *Drosophila Acon* based on the sequence lineup (Fig. 3a, Supplementary Fig. 2b, d, and Supplementary Fig. 8). The *w*^*1118*^ flies were used as the wild-type background *Drosophila*, and the heterozygous A259T KI flies (*Acon*^A259T/cyo^) were used in the present study, since the homozygous KI *Drosophila* cannot survive. Using these flies, we generated the PD model by treatment with 125 μM Rotenone (Rot) for 14 days (Fig. 3b). We showed that the ACON activity was significantly reduced in KI fly heads as compared to the *w*^*1118*^ flies at 50 days old (Fig. 3c).

Next, we tested the changes of the motor and non-motor behaviors of the KI flies treated with Rot. After treatment, the climbing capability of both the WT and KI flies was decreased, but the KI flies had a significantly more severe decline (Fig. 3d). To assay the non-motor symptoms, the DAM system was used to monitor fly activity over a 24-h period. Number of sleep episodes (number of bouts per 12 h), length of episodes (average bout length) and the total sleep length were detected. During the light phases (daytime), although the number of sleep episodes was not significantly changed (Fig. 3e), the average sleep episode length (Fig. 3f) and total sleep length (Fig. 3g) were significantly reduced in the KI flies treated or not treated with Rot. During the dark phase (nighttime), the KI flies had significantly larger number of sleep episodes than the WT flies (Fig. 3h). Moreover, the average sleep episode time was significantly decreased in the Rot-treated KI, but not WT files (Fig. 3i), while there was no significant change of total sleep length (Fig. 3j). These results suggested that the A259T variant interrupts the motor (climbing) function and aggravates the non-motor symptoms (number, average and total sleep episodes) in daytime and nighttime in the Rot-induced PD model.

Pathologically, the decreased TH protein level (Fig. 3k, l) and reduced TH-positive neurons in PPL1 and PPM1/2 cluster were detected in the heads of KI flies treated with the Rot (Fig. 3m, n), indicating the loss of DA neurons. However, the TH protein level and the number of TH^+^ neurons showed no obvious change in the KI flies without the Rot-treatment (Fig. 3m, n), indicating that the variant alone may be insufficient to induce DA neuron loss, but can lead to the locomotion defects, which is consistent with the effect of A252T variant in mice.

### ACO2 deficiency inhibits the mitochondrial function in the cell models

**Fig. 4:**
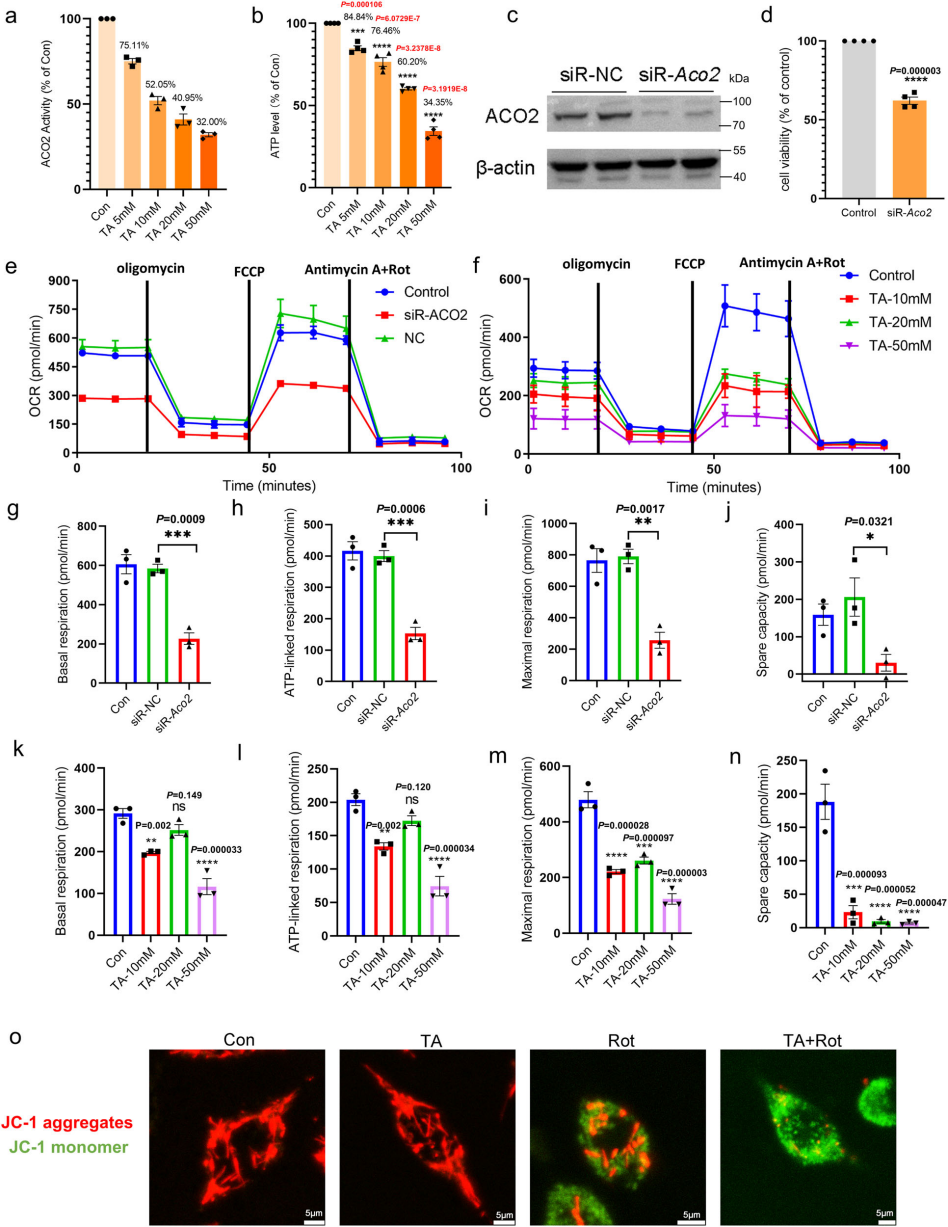
Mitochondria function is decreased in ACO2 deficient MES23.5 cells. **a** ACO2 activity in TA-treated MES23.5 cells. The data expressed as a percentage of the control (Con). *n* = 3. **b** ATP level in TA-treated MES23.5 cells. The data expressed as a percentage of the Con. *n* = 4. Results are expressed as the mean ± SEM. ****P < 0.0001 and ***P < 0.001 vs Con. Differences among means were determined by one-way ANOVA followed by the Dunnett’s test. **c** Expression levels of ACO2 in MES23.5 cells were determined by Western blotting. **d** Cell viability in siR-*Aco2*-treated MES23.5 cells measured by CCK-8 assay. The data expressed as a percentage of the Control. *n* = 4. ****P < 0.0001 vs Con. Statistical significance was performed with an unpaired *t* test between two groups. **e** The oxygen consumption rate (OCR) measured in siR-*Aco2*-treated MES23.5 cells. **f** The OCR measured in TA-treated MES23.5 cells. **g**–**j** Basal respiration, ATP-linked respiration, maximal respiration, and spare respiratory capacity are calculated from OCR data of siR-*Aco2*-treated MES23.5 cells. *n* = 3. ***P < 0.001, **P < 0.01 and *P < 0.05. Differences among means were determined by one-way ANOVA followed by the Tukey’s test. **k**–**n** Basal respiration, ATP production, maximal respiration, and spare respiratory capacity are calculated from OCR data of TA-treated MES23.5 cells. *n* = 3. ****P < 0.0001, ***P < 0.001 and **P < 0.01 vs Con. Differences among means were determined by one-way ANOVA followed by the Dunnett’s test. **o** JC-1 staining (scale bars, 5 µm). Red fluorescence: JC-1 aggregates in mitochondrial matrix; green fluorescence: JC-1 monomers distributed at cytoplasm. *n* = 3. Con: Control cells, TA: tricarballylic acid. All the *P* values were two-sided. Source data are available as a Supplementary Data 1 file.

To address the mechanisms underlying the ACO2-dependent mitochondrial regulation, we examined the oxygen consumption rate (OCR) and mitochondrial membrane potential (MMP) in the TA (aconitase 2 inhibitor)-treated or *Aco2* knockdown MES23.5 cells. The ACO2 activity and intracellular ATP level decreased with the treatment of TA in a concentration-dependent manner (Fig. 4a, b). The cell viability was also significantly inhibited by its specific siRNA (Fig. 4c, d). Additionally, as determined by the Seahorse XF system, the OCR was inhibited in cells in which the *Aco2* was blocked by siRNA (Fig. 4e) and concentration-dependent TA treatment (Fig. 4f). The basal respiration, ATP-linked respiration, maximal respiration and spare respiratory capacity were significantly decreased in cells with the *Aco2*-siRNA knockdown (Fig. 4g–j) and TA inhibition (Fig. 4k–n). As shown in Fig. 4o, JC-1(red fluorescence) was aggregated within the mitochondrial matrix, showing a normal mitochondrial membrane potential (MMP) in the control and TA-treated cells. In contrast, in Rot-induced cells, it was translocated to cytoplasm and stained as green-fluorescence monomers and with less red-aggregates in the matrix, indicating lower MMP. Furthermore, the JC-1 aggregates in mitochondrial matrix were almost completely diminished in cells with both the TA- and Rot-treatment, suggesting that the ACO2 deficiency increases the MMP decrease in Rot-induced MES23.5 cells.

We further validated these findings in cultured neurons from the *Aco2* KI mice. In the embryonic cortical primary neurons of the homozygous KI mice, the OCR was significantly decreased compared to those from the WT mice (Supplementary Fig. 9a, c–f), while there was no significant difference between those from the heterozygous KI and WT mice (Supplementary Fig. 9b, g–j). Additionally, we investigate the mitochondrial dynamics in ACO2 deficient mice. However, there was no alteration of the mitochondrial fission-related protein DRP1 and mitochondrial fusion protein MFN2 in the SN of homozygous KI mice (Supplementary Fig. 10a–d), suggesting ACO2 deficiency may not affect mitochondrial functions by disrupting mitochondrial dynamics.

### ACO2 deficiency inhibits the histone acetylation-dependent regulation of transcription of autophagy genes

**Fig. 5:**
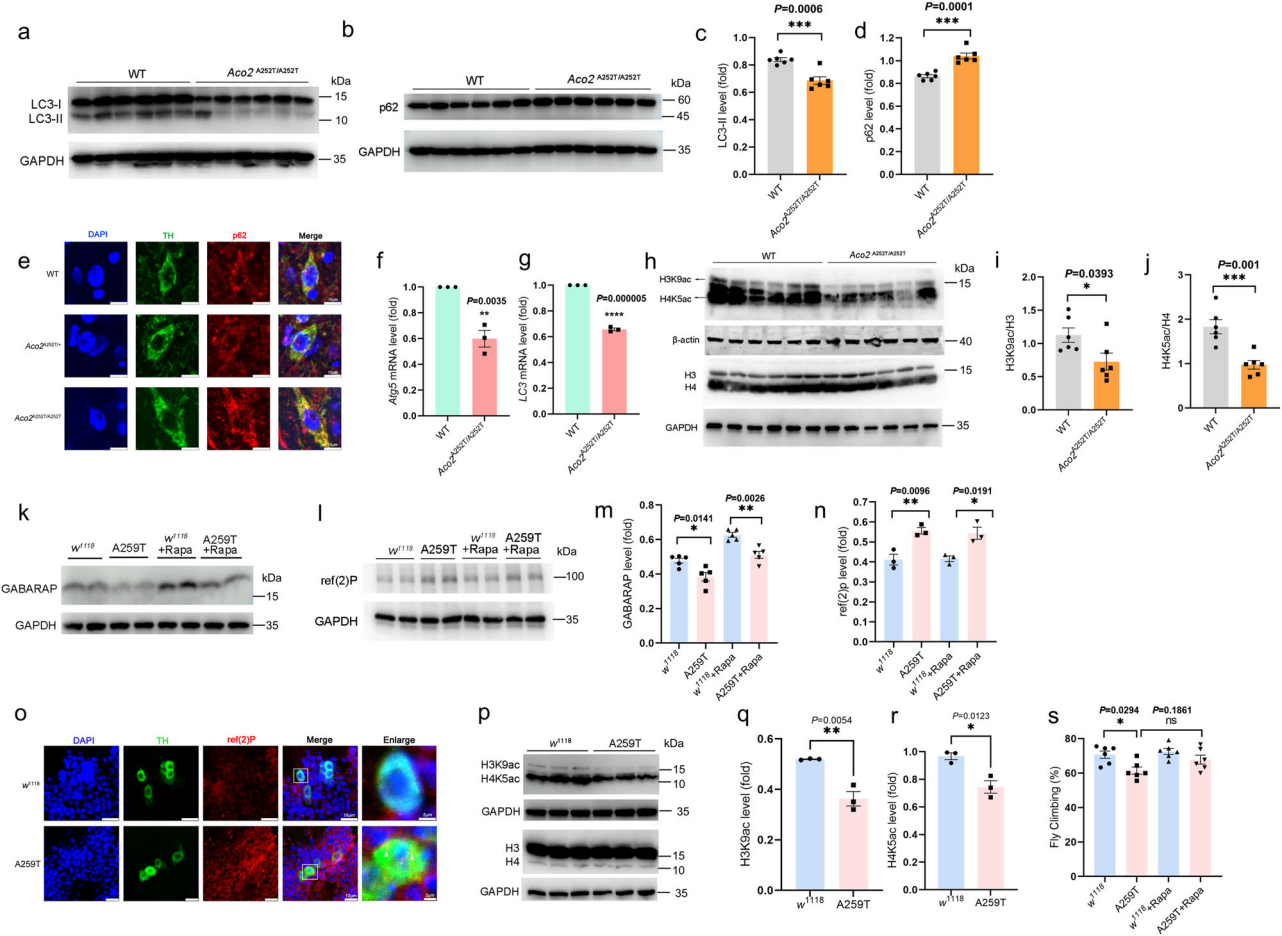
Autophagy and the histone acetylation were inhibited in the 8 months KI animals. **a**, **b** Expression levels of LC3 and p62 in the SN of mice were determined by Western blotting. **c**, **d** Quantification of LC3-II and p62 expression in the SN. *n* = 6. **e** Immunofluorescent staining of p62 (red) in the SN*pc*, TH^+^ neurons were stained in green, and the nuclei were stained with DAPI (blue) (scale bars, 10 µm). *n* = 3. **f**, **g** Quantification of *LC3* and *Atg5* mRNA expression in the SN determined by qPCR. *n* = 3. **h** Expression levels of H3K9ac and H4K5ac in the SN of mice were determined by Western blotting. H3K9ac, H4K5ac and β-actin are derived from one membrane, whereas H3/H4 and GAPDH were derived from a separate membrane. **i**, **j** Quantification of H3K9ac and H4K5ac expression in the SN. *n* = 6. **k**, **l** Expression levels of GABARAP and p62/ref(2)P in fly heads determined by Western blotting. **m**, **n** Quantification of (K and L). *n* = 3–5. **o** Immunofluorescent staining of p62/ref(2)P (red) in DA neurons (*TH* > *GFP*, green) of fly heads, the nuclei were stained with DAPI (blue) (scale bars, 10 µm, enlarged scale bars 2 µm). **p** Expression levels of H3K9ac and H4K5ac in fly heads were determined by Western blotting. **q**, **r** Quantification of H3K9ac and H4K5ac expression in fly heads. *n* = 3. **s** Climbing behavior of Rapa-treated flies. *n* = 6. Results are expressed as the mean ± SEM. ***P < 0.001, **P < 0.01 and *P < 0.05. Statistical significance was performed with an unpaired t test between two groups, and a one-way ANOVA with Tukey’s post hoc test was used for comparing more than two groups. All the *P* values were two-sided. Source data are available as a Supplementary Data 1 file.

Previous studies have shown that autophagy dysregulation causes the obstruction of α-syn degradation, misfolding and aggregation^31^, and that the decrease of histone acetylation is closely related to the transcriptional inhibition of autophagy genes^32–34^. We then asked whether the ACO2-related α-syn aggregation was due to the histone acetylation-mediated dysregulation of autophagy. We found that the expression of LC3-II and p62, the autophagosome marker and the substrate of autophagy, were dramatically decreased and increased, respectively, in the 8 month-homozygous KI mice (Fig. 5a–d). Consistently, p62 was markedly increased in the SN DA neurons of the homozygous KI mice (Fig. 5e). Moreover, the mRNA expression of *Atg5* and *LC3*, the autophagy-related genes, was significantly decreased in the SN of the homozygous KI mice (Fig. 5f, g), suggesting that the autophagy might be inhibited via autophagy-related gene transcriptions. Furthermore, we showed that the acetylation level of H3K9 and H4K5 was significantly lower in the SN of the homozygous KI than the WT mice (Fig. 5h–j). These data collectively demonstrated that the A252T variant that causes the decreased ACO2 activity may inhibit autophagy via suppressing the histone acetylation-dependent transcription of the autophagy genes.

To validate the autophagy alteration in ACON deficient flies, we detected the expression of GABARAP and p62/ref(2)P in fly heads (50 days). The GABARAP protein is a marker of autophagy that promotes autophagosome maturation, and p62/ref(2)P is the *Drosophila* homolog of the mammalian autophagy substrate p62^35^. The autophagy activator Rapamycin (Rapa) diet supplementation (200 μM in food) for *w*^*1118*^ and A259T flies was initiated after birth and continued for 50 days. Our results showed that Rapa supplementation significantly increased the GABARAP level in *w*^*1118*^ and A259T flies, and the GABARAP expression was reduced in the A259T and A259T+Rapa flies compared to the *w*^*1118*^ and *w*^*1118*^+Rapa flies (Fig. 5k, m). The p62/ref(2)P was dramatically increased in the A259T fly heads, and accumulated in the DA neurons of A259T flies (Fig. 5l, n, o), suggesting the decreased autophagic flux in ACON deficient flies. Moreover, the acetylation level of H3K9 and H4K5 was significantly reduced in the A259T fly heads (Fig. 5p–r). In addition, activating autophagy by Rapa supplementation can improve the declined motor activity of A259T flies (Fig. 5s).

**Fig. 6:**
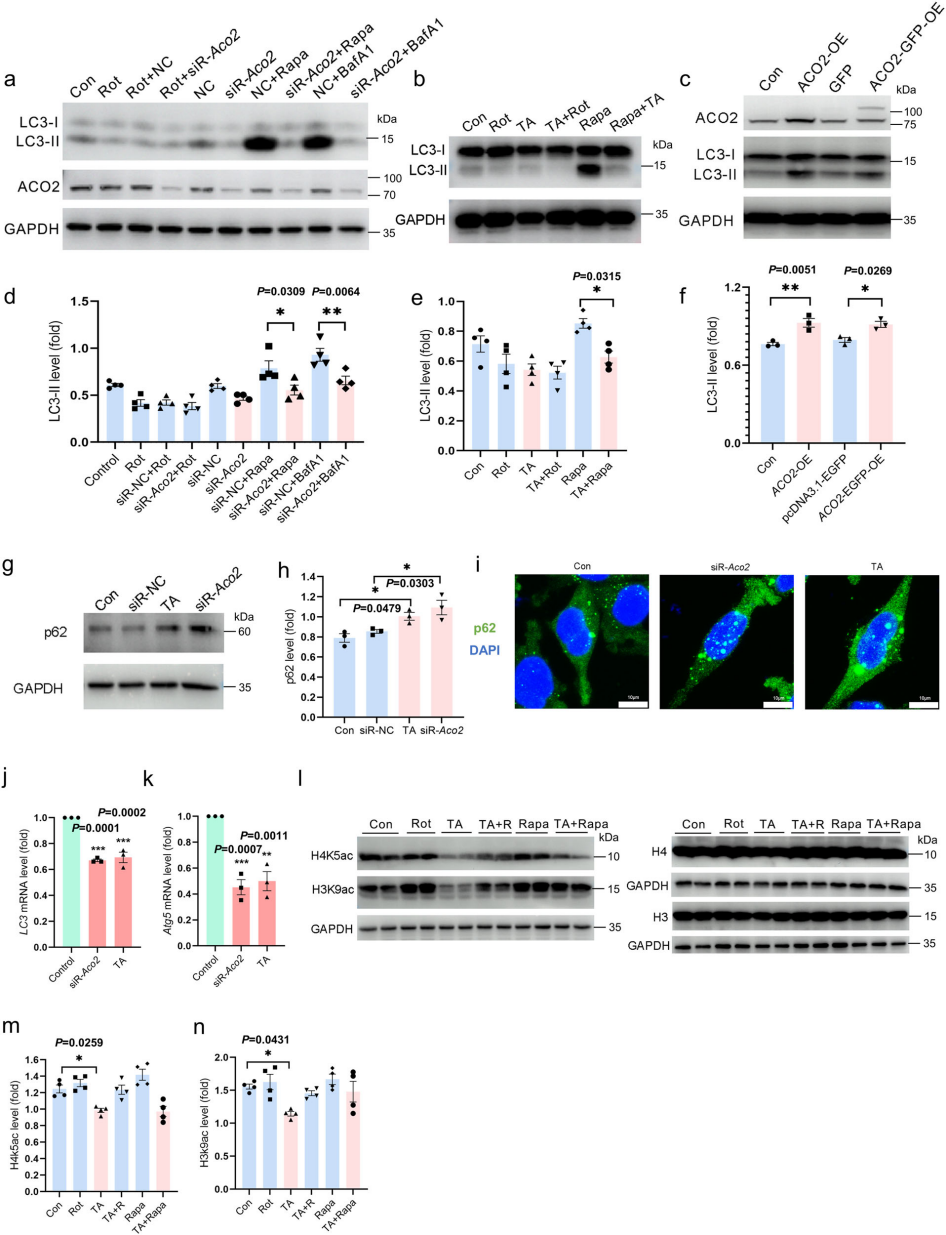
Autophagy and histone acetylation are inhibited in ACO2 deficient MES23.5 cells. **a** Expression levels of LC3 and ACO2 in *Aco2-*konckdown MES23.5 cells were determined by Western blotting. **b** Expression levels of LC3 in TA-treated MES23.5 cells were determined by Western blotting. **c** Expression levels of LC3 and ACO2 in *ACO2*-overexpressed MES23.5 cells were determined by Western blotting. pLX307-*ACO2*-V5 (*ACO2*-OE), pcDNA3.1- *ACO2*-EGFP (*ACO2*-GFP-OE) and pcDNA3.1-EGFP (GFP) were transfected in MES23.5 cells for 72 h. **d** Quantification of (**a**), *n* = 4. **e** Quantification of (**b**), *n* = 4. **f** Quantification of (**c**), *n* = 3. **g** Expression levels of p62 in MES23.5 cells were determined by Western blotting. **h** Quantification of (**g**), *n* = 3. **i** Immunofluorescent staining of p62 (green) in MES23.5 cells, nuclei were stained with DAPI (blue) (scale bars, 10 µm). *n* = 3. **j**, **k** Quantification of *LC3* and *Atg5* mRNA expression in MES23.5 cells determined by qPCR. *n* = 3. **l** Expression levels of H3K9ac and H4K5ac in TA-treated MES23.5 cells were determined by Western blotting. Since H3K9ac and H4K5ac have the same molecular weight as H3 and H4, respectively, we performed experiments on two SDS-PAGE gels separately and provide the results of two membranes. **m**, **n** Quantification of (**l**), *n* = 4. Results are expressed as the mean ± SEM. ***P < 0.001, **P < 0.01 and *P < 0.05. TA + R: TA+Rot, TA+Rapa: TA+Rapamycin. Differences among means were determined by one-way ANOVA followed by the Tukey’s test for post-hoc comparisons (**d**–**f**, **h**, **m** and **n**) or Dunnett’s test (**j** and **k**). All the *P* values were two-sided. Source data are available as a Supplementary Data 1 file.

We further validated the ACO2-mediated autophagy regulation using the ACO2-knockdown and blockade cell models. To assess the autophagic flux, cells were treated by Rapa and Bafilomycin A1 (Baf A1). The LC3-II expression was increased in both Rapa- and Baf A1-treated control cells. In contrast, its expression could not be activated by Rapa and Baf A1 in the siR-*Aco2*-knockdown cells (Fig. 6a, d). Similar results were observed in cells in which the ACO2 activity was blocked by TA (Fig. 6b, e). Consistently, the LC3-II level was increased by the ACO2 overexpression in MES23.5 cells (Fig. 6c, f). The p62 expression was increased in cells that the ACO2 was knock-downed or blocked by the *Aco2*-siRNA and TA, respectively (Fig. 6g–i). Moreover, the mRNA expression of *LC3* (Fig. 6j) and *Atg5* (Fig. 6k) the autophagy-related genes, was significantly decreased in the *Aco2*-knockdown and TA-treated cells. These changes were consistent with the significantly inhibited acetylation of H3K9 and H4K5 by the TA (Fig. 6l–n). These data suggested that the autophagic flux was dependent on the cellular ACO2 activity, which modulates the histone acetylation-based transcription of autophagy genes. Moreover, the decrease of autophagy in embryonic cortical primary neurons of the homozygous KI mice was evidenced by the decrease of LC3-II and the increase of p62 level, while there was no significant change in heterozygous KI mice compared to the WT mice (Supplementary Fig. 9k–o). However, the ACO2-dependent autophagy did not alter the other autophagy-related pathways, such as p-AMPK, p-PI3K III, p-m-TOR, ATG5 or p-Beclin 1 (Supplementary Fig. 11a–e).

In addition, for the ACO2 deficiency causes mitochondrial dysfunction and autophagy deregulation, we tested whether it disturbs mitophagy in cells. We showed that the expression of both PINK1 and Parkin, the two key markers for mitophagy, was increased in the Rot-treated MES23.5 cells, indicating the injured mitochondrial (Supplementary Fig. 12a,b). Interestingly, in the Rot-treated ACO2-deficient cells, the expression of PINK1 and Parkin was further elevated (Supplementary Fig. 12a, b). Consistently, PINK1 and Parkin were increased in the SN DA neurons of homozygous KI mice (Supplementary Fig. 12c, d). Combined with the decreased LC3-II, these preliminarily suggested that the increased dysfunctional mitochondrial may not be effectively degraded due to the inhibited autophagy in the ACO2 deficient cells.

### Coenzyme Q10 recovered the mitochondrial dysfunction and autophagy impairment in the ACO2 KI flies and TA-treated MES23.5 cells

**Fig. 7:**
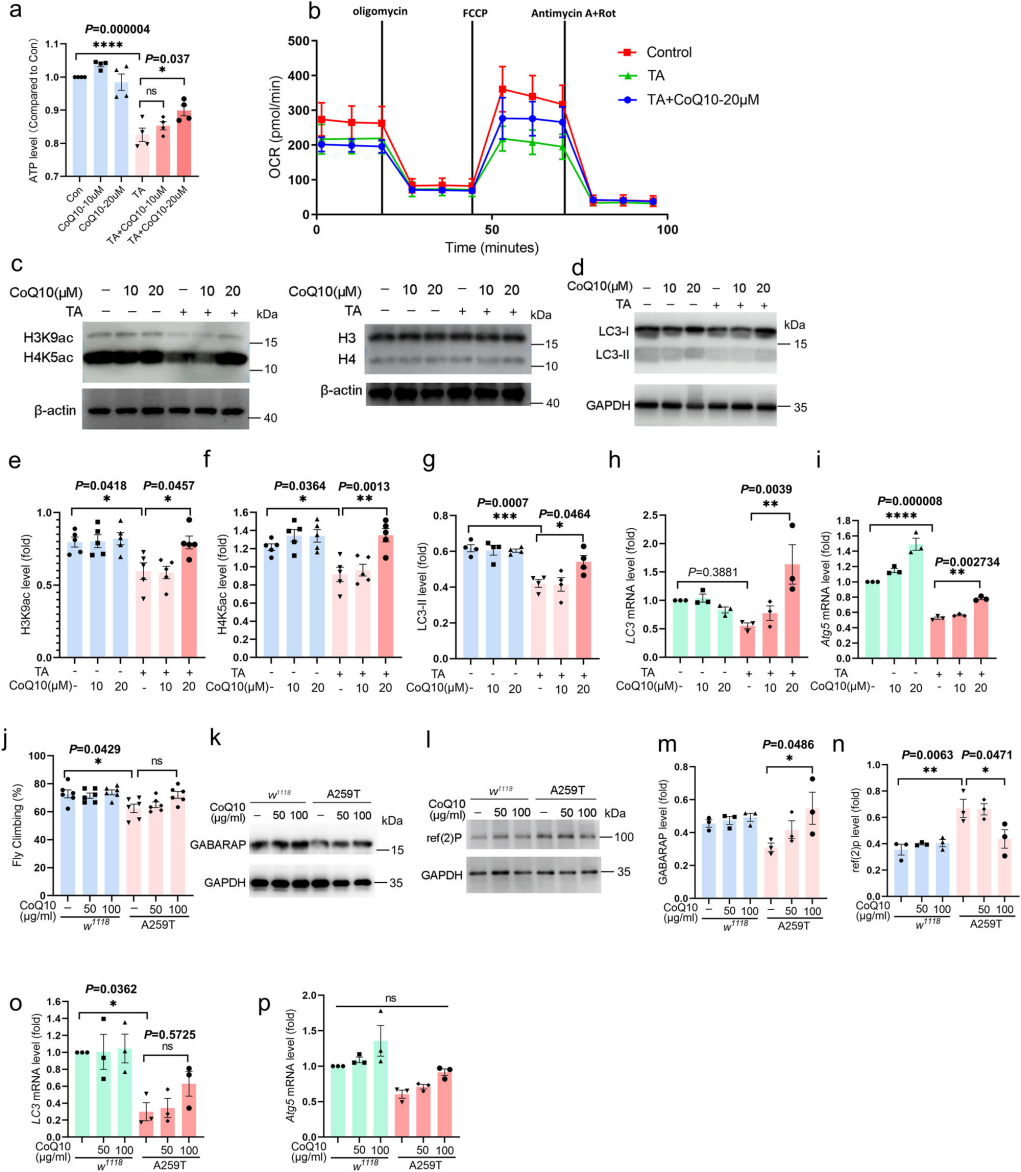
Coenzyme Q10 rescues the mitochondria function by promoting histone acetylation and autophagy in ACO2 deficient MES23.5 cells and A259T flies. **a** ATP level in MES23.5 cells. The endogenous ATP level was detected by luminescent cell viability assay, and the data expressed as a percentage of the Con. *n* = 4. **b** The OCR measured in MES23.5 cells. **c** Expression levels of H3K9ac and H4K5ac in TA-treated MES23.5 cells were determined by Western blotting. Since H3K9ac and H4K5ac have the same molecular weight as H3 and H4, respectively, we performed experiments on two SDS-PAGE gels separately and provide the results of two membranes. **d** Expression level of LC3 in TA-treated MES23.5 cells were determined by Western blotting. **e**, **f** Quantification of (**c**), *n* = 5. **g** Quantification of (**d**), *n* = 4. **h**, **i** Quantification of *LC3* and *Atg5* mRNA expression in MES23.5 cells determined by qPCR. *n* = 3. **j** Climbing behavior of CoQ10-treated flies. *n* = 6. **k**, **l** Expression levels of GABARAP and p62/ref(2)P in fly heads determined by Western blotting. **m**, **n** Quantification of (**k** and **l**). *n* = 3. **o**, **p** Quantification of *LC3* and *Atg5* mRNA expression in fly heads determined by qPCR. *n* = 3. Results are expressed as the mean ± SEM. ****P < 0.0001, ***P < 0.001, **P < 0.01 and *P < 0.05. Differences among means were determined by one-way ANOVA followed by the Tukey’s test for post-hoc comparisons. All the *P* values were two-sided. Source data are available as a Supplementary Data 1 file.

Coenzyme Q10 (CoQ10) acts as an electron carrier in the mitochondrial electron transport chain (ETC) and is an effective lipid soluble antioxidant. It can improve mitochondrial function in several PD animal models^36,37^. Firstly, we validated the CoQ10 effect on mitochondria and autophagy in MES23.5 cells. The results showed that the ATP level was significantly decreased in the TA-treated cells, which can be significantly recovered by adding CoQ10, especially at 20 μM (Fig. 7a). In addition, 20 μM CoQ10 improved the OCR level in the TA-treated cells (Fig. 7b). Interestingly, 20 μM CoQ10 can also enhance the acetylation of H3K9 and H4K5 (especially H4K5) inhibited by TA (Fig. 7c, e, f). Accordingly, the expression of the LC3-II protein (Fig. 7d, g), as well as the transcription of *LC3* (Fig. 7h) and *Atg5* (Fig. 7i), was significantly increased in the TA-treated cells when supplemented with CoQ10. However, CoQ10 did not change the level of H3K9 and H4K5 acetylation in cells not treated with TA, suggest its specific effect on the ACO2-deficient cells. Together, these data suggested that CoQ10 partially recovers the mitochondrial dysfunction and impaired autophagy by specifically regulating the ACO2-dependent histone acetylation and the transcription of autophagy-related genes.

We then tested whether CoQ10 can rescue the motor deficits caused by the ACO2 deficiency. The result showed that the fly climbing activity was improved by 100 μg/ml CoQ10 supplementation for 50 days in the KI flies, while there was no significant effect on the WT flies (Fig. 7j). Moreover, the markedly decreased GABARAP and increased p62/ref(2)P expression in the A259T files were significantly reversed by adding the 100 μg/ml CoQ10 (Fig. 7k–n). Moreover, the downregulated transcription of *LC3* and *Atg5* (Fig. 7o, p) was increased by the adding of CoQ10, although they did not reach the statistical significance. In contrast, CoQ10 have no effect on autophagy in the WT flies. These data were consistent with those observed in MES23.5 cells, indicating that the ACO2 deficiency-caused autophagy impairment may be due to the mitochondrial dysfunction.

## Discussion

**Fig. 8:**
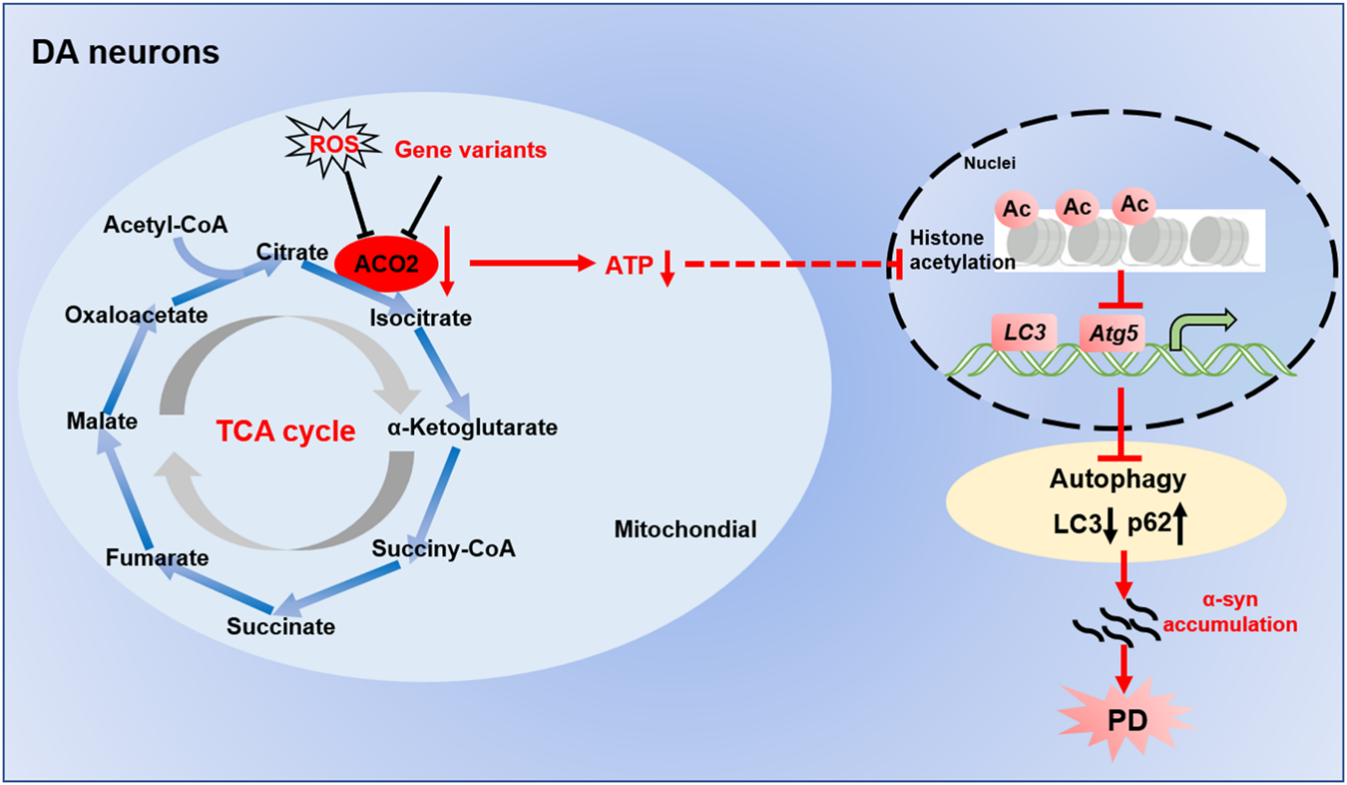
Schematic model of ACO2-mediated dysregulation of mitochondria and autophagy in PD. ACO2 deficiency leads to mitochondrial dysfunction, decrease of ATP production, decline of histone acetylation and the transcription inhibition of *LC3* and *Atg5*, resulting in inhibited autophagy and abnormal aggregated α-syn in DA neurons.

In this study, we found that the ACO2 activity was significantly decreased in the PBMCs from PD patients, and identified rare coding variants that lead to decreased aconitase activity. In the mouse and *Drosophila* KI models of the activity-reducing variant, we found aggravated motor deficits and DA neuron degeneration by the 6-OHDA and Rot induction. Moreover, we revealed that ACO2 deficiency increases the vulnerability to PD via promoting mitochondrial dysfunction and abrogating the autophagic influx through inactivation of histone acetylation-dependent transcription of autophagy-related genes. These data delineate a ACO2-mediated regulatory pathway for PD, as indicated in Fig. 8.

It has been well documented that reactive species and free radicals inactivate ACO2, rendering it a potential biomarker for oxidative damage as well as intramitochondrial sensor of redox state^38,39^. Since oxidative damages have been known as early events triggering the onset of neurodegeneration processes, it seems that inactivation and dysfunction of energy metabolism-related molecules including ACO2 due to oxidative stress could contribute to the development and promotion of various types of neurodegenerative disorders^17^. Clinical investigations have evidenced a significant decrease of ACO2 activity in PBMCs from Huntington (HD) patients and PreHD carriers^40^, as well as in patients with AD and mild cognitive impairment (MCI)^41^. Moreover, the ACO2 activity was lower in HD than PreHD, and lower in AD than MCI. Consistently, reduced levels of ACO2 activity were detected in the brains of subjects with familial or sporadic AD^42,43^. Our study further elaborated that it was significantly decreased in PBMCs from PD patients, and was positively correlated with the AAOs, and reversely associated with the disease durations. Moreover, lifestyles such as well-water drinking and exposure to pesticides further reduced the activity. These data collectively demonstrated that the PBMC ACO2 is a strong candidate biomarker indicating progression and reflecting the oxidative stress-related environmental exposures, and can be used for future clinical trials of potential antioxidants for neurodegenerative diseases. Moreover, the KI animals were more vulnerable to 6-OHDA and Rot, the well-known ROS-producing toxins. However, considering the amino acid cluster-binding site are cysteine at positions 385, 448 and 451, but not the A252T variant, we hypothesized that the KI animals were not more sensitive to peripheral and/or central exposure to ROS than the WT animals, but may be directly involved in PD pathogenesis through other pathways.

Since declined ACO2 activity reduces the OCR, damages the mitochondrial oxidative respiratory chain (decreased expression of mitochondrial complex I–IV) and inhibits mtDNA synthesis^20,44^, it is critical for the maintenance of mitochondrial function, and may play even more important roles in DA neurons with high energy requirements. We found mitochondrial dysfunction, including decreased cell viability, reduced intracellular ATP level and declined mitochondrial OCR, in the ACO2 deficient MES23.5 cells and primary neurons, without alterations of mitochondrial dynamics in ACO2 deficient mice. However, the mouse and *Drosophila* KI models with the PD patient-derived *Aco2*-A252T variant did not show significant loss of DA neurons, suggesting that the mitochondrial impairment occurs prior to the death of DA neurons. These data were consistent with the features of conditional knockout mice of *Ndufs2* (encoding the catalytic core subunit of mitochondrial complex I), in which the expression of TH semi-decreased at 60 days and motor symptoms developed at 100 days^45^. However, the KI animals showed significant behavioral impairment, which is consistent with the previous study that the ACON RNAi flies showed reduced locomotor activity^15^, indicating that the early motor performance may not be entirely dependent on the DA neuron degeneration. The ACO2 deficiency-mediated mitochondrial dysfunction have been associated with a variety of molecular mechanisms, including those dependent on acetylation-dependent activation of aconitase by sirtuin 3 (SIRT3)^25^, p53 recruitment^46^, NR1D1 regulation^47^ and so on, under different disease models. Although we provided the direct pathological evidence of increased α-syn (and p-α-syn) and DA neuron degeneration in both the mouse and *Drosophila* KI models, it remains to be elucidated the downstream molecules specifically related to these pathological changes.

We also revealed that the ACO2 depletion causes dysregulated autophagy (evidenced by LC3-II and p62 in the SN) via the regulation of transcription of *LC3* and *Atg5*, the autophagy-related genes. This is similar to the *ACO2*-overexpressed breast cancer cells that phosphorylation of Foxo1 promotes the transcription of autophagy-related genes^48^. In addition, ACON RNAi flies showed inhibition of autophagy, overexpression of *ACO2* in middle-aged flies can prolong life span and improve age-related memory impairment by increasing the expression of Atg8a and reducing the accumulation of p62^49^. Moreover, the acetylation of H3K9 and H4K5 was abolished in the SN of the ACO2 deficient mice, flies and cells, suggesting that ACO2 deficiency constitutes the autophagy dysregulation machinery via the histone acetylation-dependent modulation of gene expression. However, the acetylation of H3K9 and H4K5 in PBMCs of PD patients was increased compared to HCs (Supplementary Fig. 13), which was not consistent with the results in animal models. We assume that the epigenetic regulation in peripheral blood cells may be different from that in DA neurons in the CNS.

The molecular mechanisms underlying the mitochondrial ACO2 regulates the nuclear epigenetic modifications have been proposed. ACO2 localizes both in the mitochondria and the nucleus, owing to the N-terminal mitochondrial targeting sequence and the C-terminal nuclear localization signal (NLS)^50,51^. Previous studies have demonstrated the mitochondria-nuclear translocation^52^ and nuclear localization and operation of ACO2^53^, and that the nuclear ACO2 is crucial for the heterochromatin-associated gene silencing through interaction with Chp1^51^. In addition, the TCA cycle intermediates, such as PDHA1, PCB, ACO2, CS, IDH3A, are translocated to the nucleus during somatic cell reprogramming^54^. However, we did not detect the ACO2 signal in the nucleus of the rotenone- or TA-treated MES23.5 cells (Supplementary Fig. 14). Since most of the mitochondria-to-nucleus translocation were reported in embryonic stem cells and cancer cells, we speculate that this translocation may occurs in more premature or lower differentiated cell types. Mitochondrial metabolites can act as signaling molecules to mediate epigenetic modification and thus promote the expression of metabolic genes essential for cellular homeostasis^55^, we speculate that the ACO2-histone acetylation regulation might be modulated by TCA intermediates, like Ac-coA. However, it has been shown that the level of Ac-coA is increased in the *Acon*-RNAi flies^49^, due to Ac-coA in the nucleus is particularly important for histone modification^32^, localization of Ac-coA in different subcellular structure needs to be further investigated.

CoQ10, a lipophilic substituted benzoquinone, is an obligatory component of the respiratory chain in inner mitochondrial membrane. CoQ10 is the only endogenous lipid antioxidant and an essential factor of uncoupling protein, and it controls the permeability transition pore in mitochondria. The effects of CoQ10 supplementations have been widely tested in neurological diseases, including migraine, PD, HD, AD, amyotrophic lateral sclerosis or Friedreich´s ataxia. However, sometimes CoQ10 supplementations are more efficient in animal models of diseases than in human patients^56,57^, suggesting that its effect might be imposed in the early, but not late, clinical stages of the disease. In the current study, we revealed that that CoQ10 ameliorates the motor deficit, mitochondrial dysfunction and autophagy impairment in the ACO2-deficient animals. These data provide a molecular explanation for its therapeutic effect for PD.

In conclusion, our study provides systematic evidences supporting the correlation of ACO2 with the vulnerability to PD and the detailed molecular mechanisms underlying the disease. These findings propose ACO2 as a potential early biomarker to be used in clinical trials for assessing the effects of antioxidants in PD. Moreover, improving energy metabolism by targeting ACO2 could be considered as a potential therapeutic avenue for PD and other neurodegenerative disorders.

One major limitation of this study is that we did not provide direct evidence supporting that *LC3* and *Atg5* transcription caused by the dysregulation of histone acetylation in ACO2-deficient cells, which might be addressed by other experiments such as CHIP. In addition, further evidences need to gather for supporting the roles of ACO2-dependent dysregulation of mitophagy in PD.

## Methods

### Human subjects for genetic tests and enzyme activity assay

PD patients and healthy controls (HCs) were recruited during July 2019 and December 2021 from Xuanwu Hospital of Capital Medical University and Community Health Centres of Xinjiekou and Qinglonghu in Xicheng and Fangshan districts, respectively. The diagnosis of PD was made by at least 2 specialists in PD and movement disorders based on the MDS clinical diagnostic criteria for PD^58^. All the subjects underwent a detailed questionnaire survey and face-to-face PD assessment. The questionnaires provided basic information including sex, current age, Unified Parkinson’s Disease Rating Scale (UPDRS), and epidemiological investigation. The study was approved by the ethics committee of Xuanwu Hospital of Capital Medical University. Written informed consent was obtained from all participants.

1474 PD and 1456 HCs were recruited and genomic DNA samples were extracted from peripheral blood samples. *ACO2* variations were screened by whole-genome sequencing (WGS, 536 HC/460 PD), whole-exome sequencing (WES, 765 HC/771 PD) and panel sequencing (155 HC/243 PD), which were performed for several other research projects. Another 114 PD and 117 HCs were recruited and age- and sex-matched for measuring the ACO2 expression and enzyme activity using 10 ml of peripheral venous blood and collected in tubes containing EDTA. The primer sets for the sequencing 18 exons of human ACO2 gene are listed in Supplementary Table 1.

### Isolation of human peripheral blood mononuclear cells (PBMCs) and ELISA for ACO2 level

Human PBMCs were isolated from 10 ml human peripheral venous blood in blood collection tubes containing EDTA by density-gradient centrifugation. Briefly, the fresh human peripheral venous blood was diluted 2 times with precooled 1×PBS in 50 ml centrifugal tube, then the diluted peripheral blood was carefully and slowly added to a 15 ml centrifuge tube containing 5 ml of Ficoll reagent (Ficoll-Paque Reagent, GE Healthcare, USA), and centrifuged at 2000 rpm for 20 min at room temperature (RT), erythrocytes, PBMCs and plasma were separated due to different densities. PBMCs layer above Ficoll reagent were carefully collected and placed in a new 15 ml centrifuge tube, and 10 ml 1×PBS was added to resuspended the cells, and centrifuged at 1000 rpm for 15 min at RT, the pellet is the extracted PBMCs.

PBMCs were then lysed and homogenized in RIPA lysis buffer, and the protein concentration was measured by Pierce™ BCA Protein Assay Kit (Thermo Fisher, USA) the ACO2 expression level was measured by Aconitase ELISA kit (Signalway Antibody, USA) following the protocol recommended by manufacturer.

### ACO2 activity assay

To explore the change of enzyme activity of the ACO2 variants, the protein of wild-type (ACO2-WT) and the ACO2 variants were expressed and purified from *E.coli*. The cDNA of *ACO2*-WT and the variants were cloned into the pGEX-6P-1-GST vector (the N-terminal was attached to the GST tag). The GST-ACO2 fusion protein was expressed in BL21 competent cells (TransGen, China) on the induced condition of 0.5 mM IPTG and rotation at 20 °C for 20 h at 200 rpm. The protein of GST-ACO2-WT and ACO2 variants were purified by GST-tag Protein Purification Kit (Beyotime, China) according to the manufacturer’s protocol, and the GST tag was removed by PreScission Protease (APExBIO, USA). The protein storage solution was replaced to the assay buffer provided in the Aconitase assay kit (Abcam, UK). The protein concentration measured by Pierce™ BCA Protein Assay Kit (Thermo Fisher, USA) and the ACO2 activity was measured by Aconitase assay kit (Abcam, UK) following the protocol recommended by manufacturer.

For measuring the endogenous ACO2 activity, 1 × 10^6^ cultured cells or 50 fly heads were collected and homogenized to extracting mitochondrial, the protein concentration measured by Pierce™ BCA Protein Assay Kit and the ACO2 activity was measured by Aconitase assay kit following the protocol recommended by manufacturer.

### *Aco2*-A252T knock-in mice

*Aco2*-A252T knock-in mice (*Aco2*^A252T/+^ mice) were generated by Beijing Biocytogen company (China). All the mice were housed at a constant temperature of 23 ± 1 °C with a 12:12 h light: dark cycle, and provided with food and water ad libitum. All experimental procedures were approved by the Committee on Animal Care and Usage of Capital Medical University. Male mice (1–8 months old) were used in all experiments. All mice were genotyped via PCR analysis and Sanger sequencing of tissue biopsy samples. The genotype identification primer sequence has been provided in Supplementary Table 2.

### Unilateral 6-OHDA lesion in mice

For the 6-OHDA model in mice, pentobarbital sodium (80 mg/kg) was used as anesthesia by i.p. injection and placed in a stereotaxic frame (RWD, China). WT, heterozygous KI and homozygous KI mice at 6 months-old received two sites 2 μl infusion of vehicle solution (0.9% saline solution containing 0.02% ascorbic acid) or 6-hydroxydopamine hydrochloride (6-OHDA, 7.5 μg/ two sites) (Cayman Chemical, USA) into the right striatum at a rate of 0.5 μl/min using a 10-μl Hamilton syringe (AP: +0.6 mm; ML: +1.4 mm; DV: −3.7 mm; and AP: −0.3 mm; ML: +1.9 mm; DV: −2.9 mm). After injection, the needle remains in the last position for five minutes and then slowly exits, and the mice were resuscitated by being placed on a warm electric blanket after surgery. One month later, behavioral tests were performed. And 60 days after the 6-OHDA/saline injection, the mice were sacrificed.

### Rotarod test

Mice were trained 2 consecutive days at a speed of 10 rpm/min and 22 rpm/min for 3 times/day using an automated rotarod (5 min/ time, interval of 1 h) before testing. On the test day, mice were placed on the rotating rod at an acceleration mode (from 4 to 40 rpm/min), and their time to falling was recorded for a maximum recording time of 400 s.

### Open field test

In the open field test, mice were acclimated to the experimental room in the mouse cages for 30 min and placed into the open field for 15 min to minimize stress. Then, mice were placed in the central area of the open field and observed for 5 min by lime light 5.0 system (Actimetrics software, USA). Traveled total distance and velocity were measured. The test apparatus was thoroughly cleaned with 75% ethanol between tests with different animals.

### Western blot

The cultured cells, fly heads, substantia nigra (SN) and STR of mice were lysed and homogenized in RIPA lysis buffer supplemented with PMSF, protease inhibitor and phosphatase inhibitor (Beyotime, China), and protein concentrations were quantified by BCA method. 25 μg proteins were separated by 10% or 12.5% SDS-PAGE and transferred to a PVDF membrane. Membranes were blocked with 5% non-fat dry milk in TBST for one hour at RT and incubated with primary antibodies overnight at 4 °C against specific antibodies. The immunoreactive bands were visualized by a chemiluminescence system (Vilber Fusion FX7, French). Analyses were performed using the ImageJ software. Band intensities were quantified at different exposures, depending on the protein.

Antibodies: anti-TH antibody (1:1000, Millipore), anti-α-synuclein antibody (1:1000, CST), anti-LC3B antibody (1:1000, proteintech), anti-p62 antibody (1:2000, Abcam), anti-H3K9 antibody (1:5000, Active motif), anti-H4K5 antibody (1:5000, Millipore), anti-H3 antibody (1:1000, CST), anti-H4 antibody (1:1000, CST), anti-GABARAP antibody (1:1000, Abcam), anti-ref(2)P antibody (1:500, Abcam), anti-p-AMPK antibody (1:1000, Abcam), anti-p-ULK1 antibody (1:1000, CST), anti-PI3K III antibody (1:1000, CST), anti-p-mTOR antibody (1:1000, CST), anti-p-PI3K antibody (1:1000, CST), anti-ATG5 antibody (1:1000, Abcam) and anti-p-Beclin1 antibody (1:1000, CST), anti-DRP1 antibody (1:2000, proteintech), anti-MFN2 antibody (1:1000, Abcam), anti-β-actin antibody (1:5000, proteintech), anti-GAPDH antibody (1:5000, proteintech), peroxidase-conjugated anti-mouse or anti-rabbit IgG (1:5000, Zhongshan Golden Bridge Biotechnology).

### Immunofluorescence staining

For mouse brain and fly heads immunofluorescence staining, the OCT embedded mouse brains were cut into 20-μm coronal slices by freezing microtome (Leica), and the slices and extracted fly heads were fixed with 4% PFA for 20 min, and then permeabilizated by 0.3% Triton X-100 for 30 min, followed by blocking with 5% BSA for 1 h at RT. Then, the brain slices were incubated with corresponding primary antibodies overnight at 4 °C. The next day, brain slices were incubated with fluorescent-labeled secondary antibodies (1:500, Thermo Fisher, USA) and DAPI (Solarbio, China) to stain nuclei. For cultured cells immunofluorescence staining, cells were fixed with 4% PFA for 20 min, and then permeabilizated by 0.3% Triton X-100 for 10 min, followed by blocking with 5% BSA for 1 h at RT. Next, cells were incubated overnight at 4 °C with primary antibodies, and incubated with fluorescent-labeled secondary antibodies and DAPI to stain nuclei. The immunofluorescence was visualized by SP8 confocal microscope (Leica Microsystems, Germany).

Primary antibodies: anti-TH antibody (1:500, Millipore), anti-α-synuclein antibody (1:1000, CST), anti-p62 antibody (1:500, Abcam), anti-ref(2)P antibody (1:100, Abcam), anti-MAP2 antibody (1:500, Abcam), anti-p-α-synuclein antibody (1:500, Abcam), anti-PINK1 antibody (1:200, proteintech), anti-Parkin antibody (1:200, proteintech).

### Cell culture (cell line and primary neurons) and transfection

MES23.5 mouse dopaminergic neuron cell line were purchased from the Fenghui Biotechnology (Hunan Fenghui Biotechnology, China) and cultured in Dulbecco’s modified Eagle’s medium (DMEM) (Gibco, USA) with 10% fetal bovine serum (FBS) (Gibco, USA) and 100 U/ml penicillin/streptomycin (Gibco, USA) under a humidified atmosphere containing 5% CO_2_ at 37 °C.

Primary cortical neurons were extracted from embryos of mice at 13–15 days of gestation. Firstly, we isolated the cortex from the embryonic mouse brain and stripped the meninges and blood vessels covering the cortex. Next, the isolated cortices were digested by 0.25% trypsin (Gibco, USA) for 10 min in 37 °C, and suspended to single-cell in total medium (DMEM + 10%FBS + 1%PS). The cortical neurons were plated in poly-D-lysine (Sigma, USA) coating 24-well plate (4 × 10^5^ cells/well) and XFe 24-well plates (Seahorse Bioscience, USA) (1 × 10^5^ cells/well). After 6 h, the medium was replaced to Neurobasal culture medium supplemented with 2% B27 (Gibco, USA), 1%Glutamax (Gibco, USA) and 1%PS (Gibco, USA). 48 h later, 10 μM cytosine arabinoside was added to inhibit the growth of glial cells. Primary cortical neurons were cultured in a 37 °C incubator with 5% CO_2_, and the Neurobasal culture medium was changed by half every 2 days.

The ribo*FECT*^TM^ CP Reagent (RIBOBIO, China) was used for siRNA transfection, and Lipo8000 (Byotime) was used for plasmid (pLX307-*ACO2*-V5, pcDNA3.1- *ACO2*-EGFP and pcDNA3.1-EGFP) overexpression. The siRNA interference sequence has been provided in Supplementary Table 3.

### ATP level measurement and cell counting Kit-8 (CCK-8) assay

ATP level in cultured cells was measured by ATP assay kit (Promega, USA). The cells cultured in 6-well plates were lysed in ATP lysis, the supernatant was collected at 12,000 rpm and centrifuged at 4 °C for 30 min. The protein concentration was detected by BCA method. Add 50 µL sample and 50 µL ATP detection working buffer to each well of opaque 96-well white plate, and incubate at room temperature for 10 min. The luminescence value was measured by Microplate Reader. The cell viability of MES23.5 cells was detected via CCK-8 assay kit (Dojindo, Japan). 10 µL CCK-8 reagent was added to each well of the 96-well plate and incubated at 37 °C for 1.5–2 h. The absorbance at 450 nm was detected by a Microplate Reader.

### Mitochondrial stress measured by XFe-24 Seahorse assays

A Seahorse XFe analyzer (Seahorse Bioscience, USA) was used to measure the oxygen consumption rate (OCR). MES23.5 cells and mouse primary cortical neurons were seeded in XFe 24-well plates (poly-D-lysine coating for primary cortical neurons). Assay medium was prepared by supplementing Seahorse XFeBase Medium minimal DMEM (Seahorse Bioscience, USA) with 2 mM L-glutamine, 1 mM pyruvate and 10 mM glucose (Sigma, USA) for a Mito Stress Test assay (pH = 7.4). Probes (Seahorse Bioscience, USA) were hydrated in the 1 ml calibrant (Seahorse Bioscience, USA) at 37 °C incubator overnight. The cell culture medium was replaced to assay medium and kept in a non-CO_2_ incubator at 37 °C for 45 min before test. MES23.5 cells sequentially treated with 1.5 µM Oligomycin, 2 µM FCCP and 0.5 µM Antimycin A+Rot in the process of analysis, while 1.5 µM Oligomycin, 1 µM FCCP and 0.5 µM Antimycin A+Rot were sequentially treated to mouse primary cortical neurons.

### JC-1 staining

MES23.5 cells were cultured in DMEM total medium with TA (20 mM) for 48 h in confocal dish, followed by 0.5 µM Rot treatment for 24 h, and cells were stained with JC-1 fluorescent probes solution (1×) for 30 min at 37 °C. Subsequently, the cells were washed with 1×PBS and visualized by SP8 confocal microscope (Leica Microsystems, Germany).

### *Drosophila* Stocks and drug administration

*w*^*1118*^, *TH-gal4*, *UAS-GFP* flies were purchased from Bloomington Fly Stock Center, *Acon*-A259T (*Acon*^A259T/cyo^) knock-in flies were generated by Qidong Fungene Biotechnology (China). The *w*^*1118*^ and *Acon*-A259T flies were used for drug administration, behavioral tests, Western blot, qRT-PCR and TH immunofluorescence. *TH* > *GFP* and A259T/*UAS-GFP*; *TH-gal4* Flies were used for immunofluorescence staining of p62/ref(2)P. Flies were raised at 25 °C with 60% humidity under a 12 h light/dark cycle. Male flies were used in all experiments. The genotype identification primer sequence has been provided in Supplementary Table 2.

125 µM Rotenone (Rot) (MCE, China) were mixed in cornmeal fly food, the *w*^*1118*^ and A259T flies were fed from day 36 for 14 days. 200 µM Rapamycin (MCE, China) and coenzyme Q10 (CoQ10, 50 or 100 µg/ml) (MCE, China) were mixed in cornmeal fly food, the flies were fed from day 1 for 50 days.

### Climbing ability

*w*^*1118*^ and A259T flies were gently tapped to the bottom of the container and allowed to climb up the line (13 cm) within 25 s to assay their climbing ability. The percentage of flies climbing higher than 13 cm was calculated for each genotype, flies were examined three times per experiment.

### *Drosophila* Activity Monitoring (DAM) system

The DAM contains 32 channels, each connected to a small glass tube, and the activity of individual flies can be monitored as they move back and forth along the container to passing through the infrared beam. Movements were recorded in 1 min bins and sleep (or rest) is defined as a bout of 5 or more minutes of inactivity^59^.

### Quantitative real-time reverse transcriptase polymerase chain reaction (qRT-PCR)

Total RNA was extracted from fly heads, SN of mouse brain and cultured cells by TRIzol reagent (Invitrogen, USA) with concentrations of total RNA were measured by Nanodrop spectrophotometer. The isolated RNAs were subjected to reverse transcription and polymerization reaction to obtain cDNA using the TransScript® II first strand cDNA synthesis supermix (Transgen, China) according to the manufacturer’s instructions. We conducted qRT-PCR in 96-well blocks using the realtime PCR system (Thermo Fisher, USA). The final reaction volume was 10 µl. Each reaction mix contained 2×PowerUpTM SYBRTM Green Supermix (Thermo Fisher, USA) 5 µl, 10 µM Forward Primer 0.5 µl, 10 µM Reverse Primer 0.5 µl, 15 ng/µl cDNA 1 µl and ddH_2_O 3 µl. The amplification condition included a pre-denaturation step at 95 °C for 30 s, which was followed by 40 cycles of 95 °C for 10 s and 60 °C for 30 s. After the cycling process, the melting curves of qRT-PCR amplifications were obtained by raising the temperature from 60 °C to 95 °C. The primers were designed and synthesized by Shanghai Biotechnology (China). The primer sets for the qRT-PCR are listed in Supplementary Table 4.

### Statistics and reproducibility

Statistical tests were performed using GraphPad Prism 9.0 (GraphPad Software, CA) or SPSS via two‐tailed unpaired *t* test between two groups and one-way ANOVA with Tukey’s post hoc test or Dunnett’s test for multiple comparisons. Correlations of clinical variables with ACO2 activity of PBMCs were examined by simple linear regression models. Phylogenetic and molecular evolutionary analyses were conducted using MEGA version 11. All data were obtained by performing at least 3 independent experiments with representative data shown and expressed as the mean ± standard error of the mean (SEM), with the statistical significance level set at *P* < 0.05.

### Reporting summary

Further information on research design is available in the Nature Portfolio Reporting Summary linked to this article.

### Supplementary information

Supplementary information

Description of Additional Supplementary Files

Supplementary Data 1

Reporting Summary

## Data Availability

All materials and data are available upon request. All relevant data generated for this study are available within the Article, Supplementary Information (Supplementary Figs. 15–27) and Supplementary Data 1 file. Source data are provided in the Supplementary Information (Supplementary Figs. 15–27) and Supplementary Data 1 file. The raw sequence data reported in this paper have been deposited in the Genome Sequence Archive (Genomics, Proteomics & Bioinformatics 2021) in National Genomics Data Center (Nucleic Acids Res 2022)^60,61^, China National Center for Bioinformation / Beijing Institute of Genomics, Chinese Academy of Sciences (GSA-Human: HRA005774, HRA005403 and HRA005554) that are publicly accessible at https://ngdc.cncb.ac.cn/gsa-human. All other data are available from the corresponding author (or other sources, as applicable) on reasonable request.
