# Supplementary material for: ACO2 deficiency increases vulnerability to Parkinson’s disease via dysregulating mitochondrial function and histone acetylation-mediated transcription of autophagy genes
Source: Commun Biol. 2023 Nov 25;6:1201. doi: 10.1038/s42003-023-05570-y (PMC10676364; doi:10.1038/s42003-023-05570-y)

SUPPLEMENTARY INFORMATION

Supplementary Table 1 Primers for sequencing 18 exons of human ACO2 gene

Exon number	Primer sequence
EX1-F	5’-TGTTCGTTGCACGTGAGCT-3’
EX1-R	5’-GGACAGGTACACGAGAAGTTG-3’
EX2-F	5’-GAGGCTGCAGTTACTGAACA-3’
EX2-R	5’-ACCTTGACCCTGCCACTATCA -3’
EX3-F	5’-GTTGAGGTTGCCACATGGACT-3’
EX3-R	5’-TGGTTTGCTCCAGTGTGAG -3’
EX4-F	5’-AGAAGGTCTCTAAGAGAGGG-3’
EX4-R	5’-GATGCTTGAGTACACACCCA -3’
EX5-6-F	5’-CTGCTCCTACCAGTTCCAT-3’
EX5-6-R	5’-TACTCCCAACTGGAGACCCAT -3’
EX7-F	5’-ACTCCCTGGTGTGAAGATGCA-3’
EX7-R	5’-GCTCCAGACAATCTACTCAG -3’
EX8-F	5’-CACTAGGGTGTGAGCTCTGTA-3’
EX8-R	5’-TCCCCACTACTCTCAGAAAC-3’
EX9-F	5’-CTTCAAGCACAGCATGTC-3’
EX9-R	5’-AGGTGATTGTCTACAGCCC -3’
EX10-11-F	5’-TCGGTTGCTTGCAACTGGT-3’
EX10-11-R	5’-CTGGAGTCTCTTCTCTCCA-3’
EX12-F	5’-GTGATCCTGAGGTTCCCTT-3’
EX12-R	5’-CTGGCTACCACTTGGATGA -3’
EX13-14-F	5’-TTGGCCTAGGCTTTTGGTAGG-3’
EX13-14-R	5’-TGCGTCTTGACTGTGCTCACT-3’
EX15-F	5’-CAGAACACGTGTCTGAAGAC-3’
EX15-R	5’-TTCAGGAGTGACCACTGGCATT -3’
EX16-F	5’-ATTCACGCAGGCTTCACTTGC-3’
EX16-R	5’-GCACTGTGTGAGCCTAAGAA-3’
EX17-F	5’-AAGCTCTCCAGGCTAGTCA-3’
EX17-R	5’-GCCTTTCCAGCTGGAATGAAG -3’
EX18-F	5’-AAGGGCACACAGTACCCACCA-3’
EX18-R	5’-AGTGATGGATCCATCTGTGGCTG -3’

F: Forward; R: Reverse

Supplementary Table 2 Genotype identification primer sequence

Primer	Sequence
M-Aco2-F	5’-TTTGTGAGGAGTAGGACTGAAGAAG-3’
M-Aco2-R	5’-TTTTCAGTTGTTCCATCAGTTCAGC-3’
D-Acon-F	5’-ATTGGCGTTGGTGGTGCTGATG-3’
D-Acon-R	5’-CTTGAACCAGCCGATCTGTAGGT-3’

M: Mouse; D: *Drosophila*; F: Forward; R: Reverse

Supplementary Table 3 siRNA interference sequence

siRNA	Sequence
siRNA-*Aco2*	5’-GATATGACCTGCTAGAGAA-3’

Supplementary Table 4 Primers for the qRT-PCR of autophagy-related genes

Primer	Sequence
M-*Actb*-F	5’ CTACCTCATGAAGATCCTGACC 3’
M-*Actb*-R	5’ CACAGCTTCTCTTTGATGTCAC 3’
M- *LC3*-F	5’ CTGTCCTGGATAAGACCAAGTT 3’
M- *LC3*-R	5’ GTCTTCATCCTTCTCCTGTTCA 3’
M- *Atg5*-F	5’ CCAATGCTGGAAACCCCTCT 3’
M- *Atg5*-R	5’ GGCTGGGGGACAATGCTAAT 3’
D-*Gapdh*-F	5’ TTCTGGCATTTCGCTGAA 3’
D-*Gapdh*-R	5’ CCGAACTCGTTGTCGTACCA 3’
D-*LC3*-F	5’ TCCAATACAAGGAGGAGCACG 3’
D-*LC3*-R	5’ TTGGGAGCCTTCTCGACGAT 3’
D- *Atg5*-F	5’ CAGGATGGAGCTGTGTGGTT 3’
D- *Atg5*-R	5’ CCGGATGCAGCAGATCGTAT 3’

M: Mouse; D: *Drosophila*; F: Forward; R: Reverse

Supplementary Figures

Supplementary Figure 1. Correlations between ACO2 activity in PBMCs of PD patients and scores for motor and non-motor symptoms of PD.

(a) H&Y stages. Error bars indicate mean±SEM. (b) UPDRS III Score (Unified Parkinson's disease Rating Scale part III) (motor); (c) MMSE Score (Mini-mental State Examination), MoCA Score (Montreal Cognitive Assessment). (d) AHRS Score (Hyposmia rating scale). (e) RBDQ-HK Score (Rapid eye movement sleep behavior disorder questionnaire-Hong Kong), RBDSQ Score (RBD Screening Questionnaire). (f) GDS Score (Geriatric depression scale), HAMD Score (Hamilton Depression Scale). (g) HAMA Score (Hamilton Anxiety Scale). Correlations were investigated using simple linear regression.

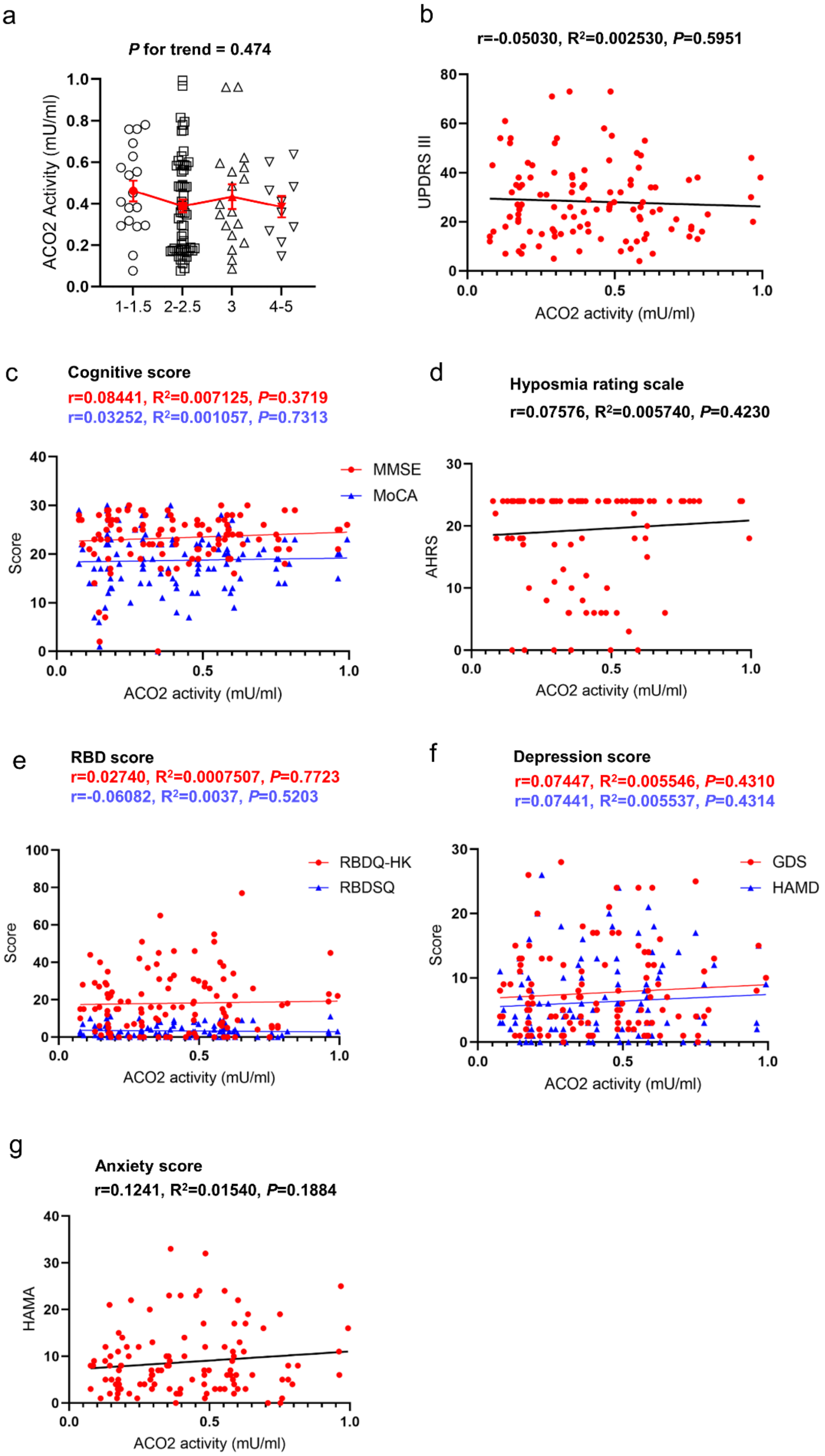
a
P for trend = 0.474
ACO2 Activity (mU/ml)
1.0
0.8
0.6
0.4
0.2
0.0
1-1.5
2-2.5
3
4-5
b
r=-0.05030, R2=0.002530, P=0.5951
UPDRS III
80
60
40
20
0
0.0
0.5
1.0
ACO2 activity (mU/ml)
c
Cognitive score
r=0.08441, R2=0.007125, P=0.3719
r=0.03252, R2=0.001057, P=0.7313
MMSE
MoCA
Score
40
30
20
10
0
0.0
0.5
1.0
ACO2 activity (mU/ml)
d
Hyposmia rating scale
r=0.07576, R2=0.005740, P=0.4230
AHRS
30
20
10
0
0.0
0.5
1.0
ACO2 activity (mU/ml)
e
RBD score
r=0.02740, R2=0.0007507, P=0.7723
r=-0.06082, R2=0.0037, P=0.5203
RBDQ-HK
RBDSQ
Score
100
80
60
40
20
0
0.0
0.5
1.0
ACO2 activity (mU/ml)
f
Depression score
r=0.07447, R2=0.005546, P=0.4310
r=0.07441, R2=0.005537, P=0.4314
GDS
HAMD
Score
30
20
10
0
0.0
0.5
1.0
ACO2 activity (mU/ml)
g
Anxiety score
r=0.1241, R2=0.01540, P=0.1884
HAMA
40
30
20
10
0
0.0
0.5
1.0
ACO2 activity (mU/ml)

Supplementary Figure 2. Identification of the heterozygous A252T variation in ACO2 of PD patients, and generation of *Aco2*-A252T mice and *Acon*-A259T flies. (a) Conservation of the A252 site between human and mouse of ACO2 protein sequence. (b) Conservation of the A252 site between human and drosophila melanogaster of ACO2 (ACON) protein sequence. (c) The sequence of the heterozygous G754A variation identified in PD patients. (d) Sequencing confirmation of G775A in flies. (e and f) Sequencing confirmation of heterozygous and homozygous G754A in mice.

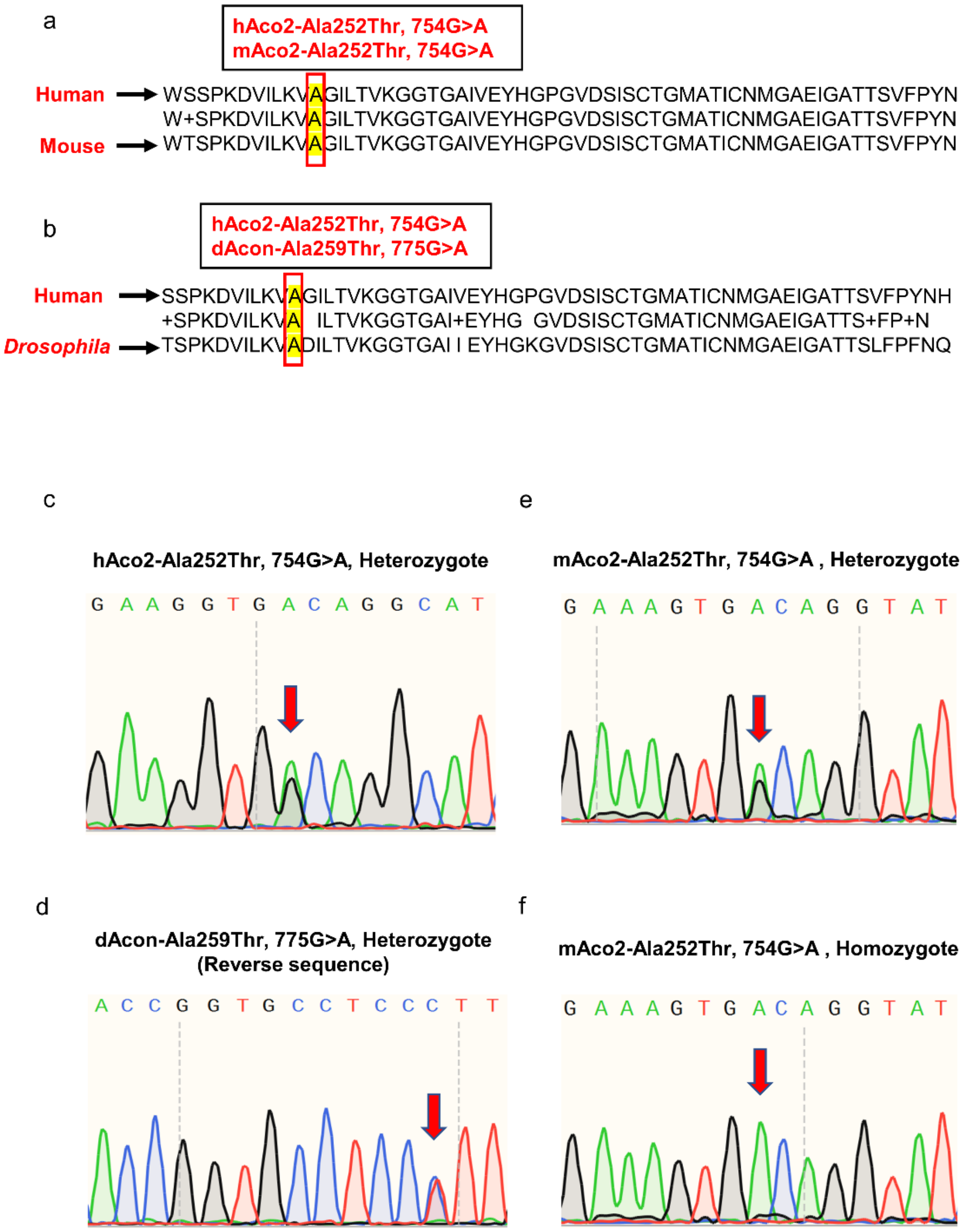
a
hAco2-Ala252Thr, 754G>A
mAco2-Ala252Thr, 754G>A
Human
WSSPKDVILKVAGILTVKGGTGAIVEYHGPGVDSISCTGMATICNMGAEIGATTSVFPYN
W+SPKDVILKVAGILTVKGGTGAIVEYHGPGVDSISCTGMATICNMGAEIGATTSVFPYN
Mouse
WTSPKDVILKVAGILTVKGGTGAIVEYHGPGVDSISCTGMATICNMGAEIGATTSVFPYN
b
hAco2-Ala252Thr, 754G>A
dAcon-Ala259Thr, 775G>A
Human
SSPKDVILKVAGILTVKGGTGAIVEYHGPGVDSISCTGMATICNMGAEIGATTSVFPYNH
+SPKDVILKVA ILTVKGGTGAI+EYHG GVDSISCTGMATICNMGAEIGATTS+FP+N
Drosophila
TSPKDVILKVADILTVKGGTGAI I EYHGKGVDSISCTGMATICNMGAEIGATTSLFPFNQ
c
hAco2-Ala252Thr, 754G>A, Heterozygote
G A A G G T G A C A G G C A T
e
mAco2-Ala252Thr, 754G>A , Heterozygote
G A A A G T G A C A G G T A T
d
dAcon-Ala259Thr, 775G>A, Heterozygote
(Reverse sequence)
A C C G G T G C C T C C C T T
f
mAco2-Ala252Thr, 754G>A , Homozygote
G A A A G T G A C A G G T A T

Supplementary Figure 3. TH level in the STR of mice in different 6-OHDA concentration.

(a) Western blot of TH. (b) Quantification of (a). n=3. Results are expressed as the mean±SEM. Statistical significance was performed with a one-way ANOVA with Dunnett's multiple comparisons test was used for comparing more than two groups. All the *P* values were two-sided. Source data are available as a Supplementary Data 1 file.

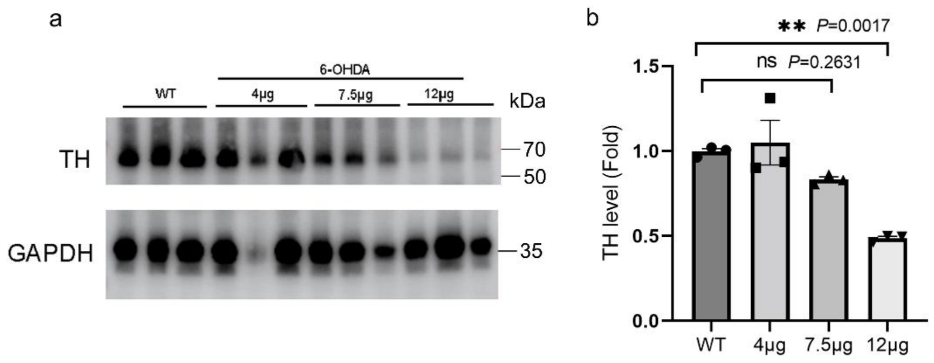

Supplementary Figure 4. Body weight of mice after 6-OHDA treatment for 30 days. 7-month mice were used for weighing the body weight, n=5-6. Results are expressed as the mean±SEM. Statistical significance was performed with a one-way ANOVA with Turkey's test. All the *P* values were two-sided. Source data are available as a Supplementary Data 1 file.

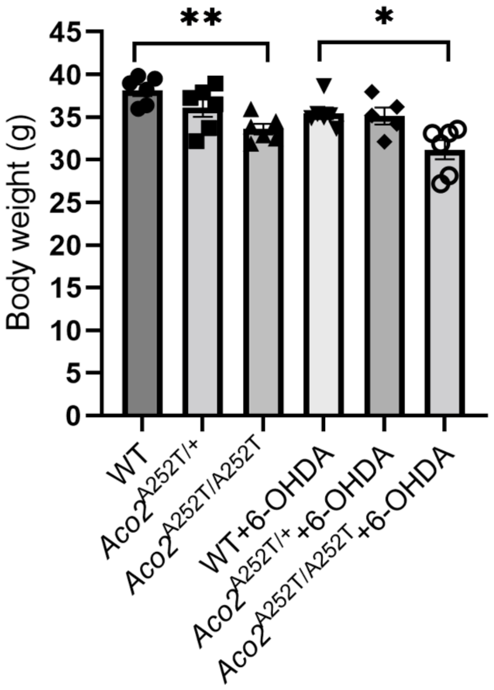

Supplementary Figure 5. TH level in the STR of mice after 6-OHDA injection for 1 month.

(a) Western blot of TH. (b) Quantification of (a).

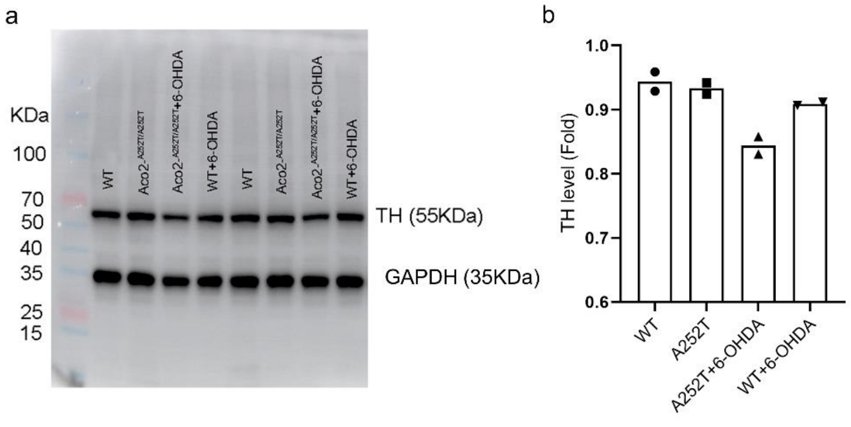

Supplementary Figure 6. TH expression in 6-OHDA-induced WT and *Aco2*$^{A252T/+}$ mice.

(a and b) Expression levels of TH in the SN and STR of WT+6-OHDA and *Aco2*$^{A252T/+}$+6-OHDA groups were determined by western blotting, respectively. (c and d) Quantification of TH expression in the SN and STR. n=6. Results are expressed as the mean±SEM. Statistical significance was performed with an unpaired t-test between two groups. All the *P* values were two-sided. Source data are available as a Supplementary Data 1 file.

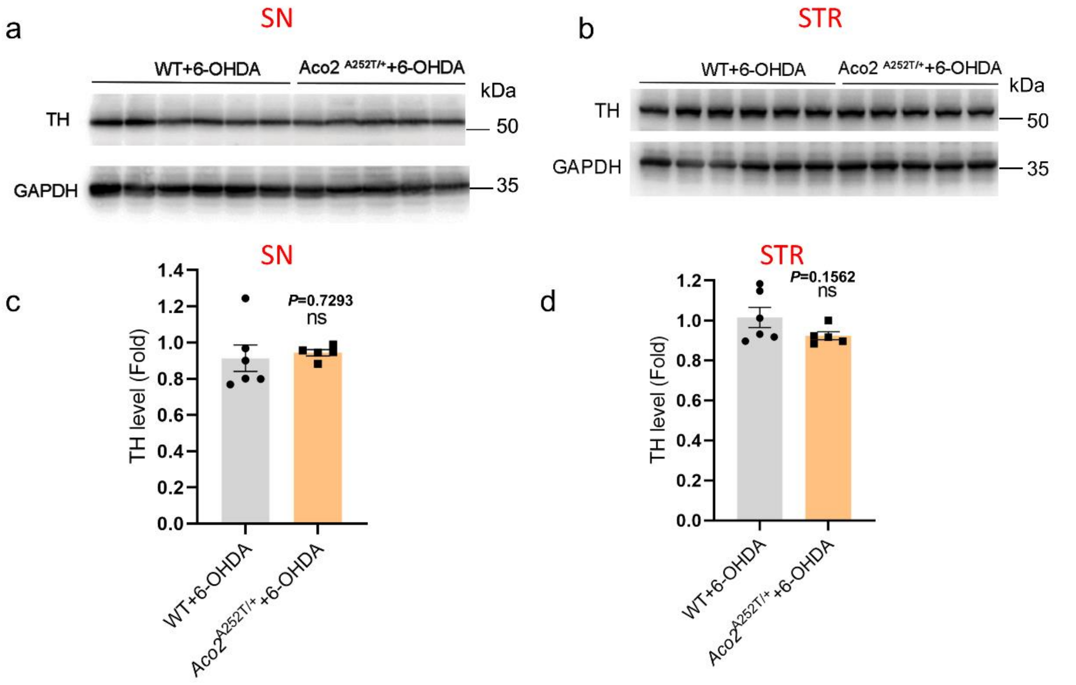

Supplementary Figure 7. Increased p-α-synuclein in the DA neurons of *Aco2*$^{A252T/A252T}$ mice.

Immunofluorescent staining of p-α-synuclein (red) in the SN*pc*, TH^{+} neurons were stained in green, and the nuclei were stained with DAPI (blue) (scale bars: 10μm, enlarged scale bars: 2μm). n=3. The white arrows: p-α-synuclein expressed in TH-positive neurons.

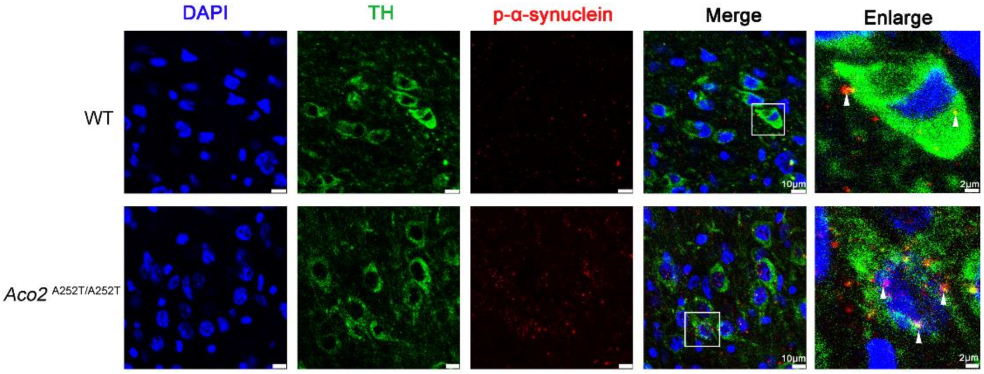

Supplementary Figure 8. The phylogeny tree was constructed to illustrate the evolutionary conservation of ACO2.

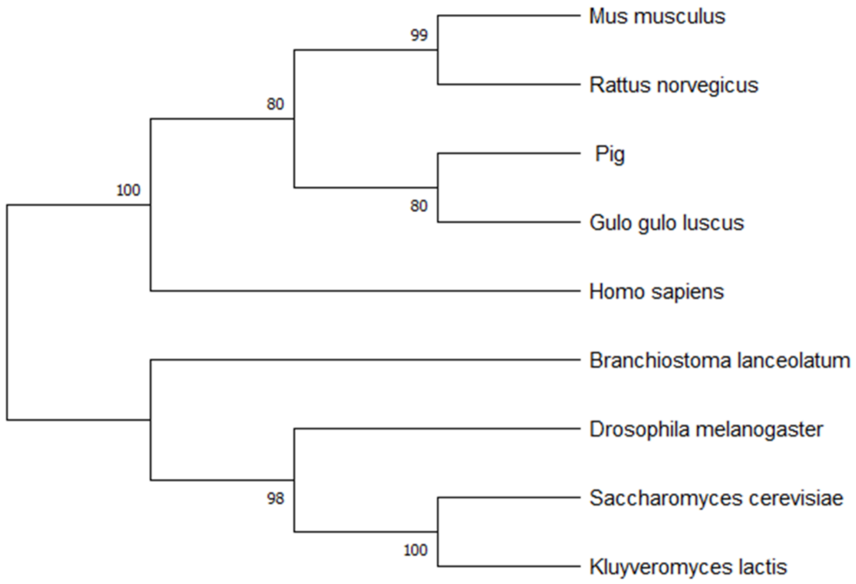

Supplementary Figure 9. Decreased mitochondrial function and autophagy in *Aco2*$^{A252T/A252T}$ mouse embryonic cortical primary neurons.

(a) The OCR measured in WT and homozygous KI mouse embryo primary cortical neurons. (b) The OCR measured in WT and heterozygous KI mouse embryo primary cortical neurons. (c-f) Basal respiration, ATP production, maximal respiration, and spare respiratory capacity are calculated from OCR data of (a). n=4. (g-j) Basal respiration, ATP production, maximal respiration, and spare respiratory capacity are calculated from OCR data of (b). n=4. (k and l) Expression levels of LC3 and p62 in mouse embryo primary cortical neurons were determined by western blotting, respectively. (m and n) Quantification of (k and l). n=3. (o) Immunofluorescent staining of p62 (green) in the primary cortical neurons, neurons stained with MAP2 (red), and the nuclei were stained with DAPI (blue) (scale bars, upper, 45μm). n=3. Results are expressed as the mean±SEM. ****P<0.0001, ***P<0.001 and **P<0.01. Statistical significance was performed with an unpaired t-test between two groups, and a one-way ANOVA with Dunnett's multiple comparisons test was used for comparing more than two groups. All the P values were two-sided. Source data are available as a Supplementary Data 1 file.

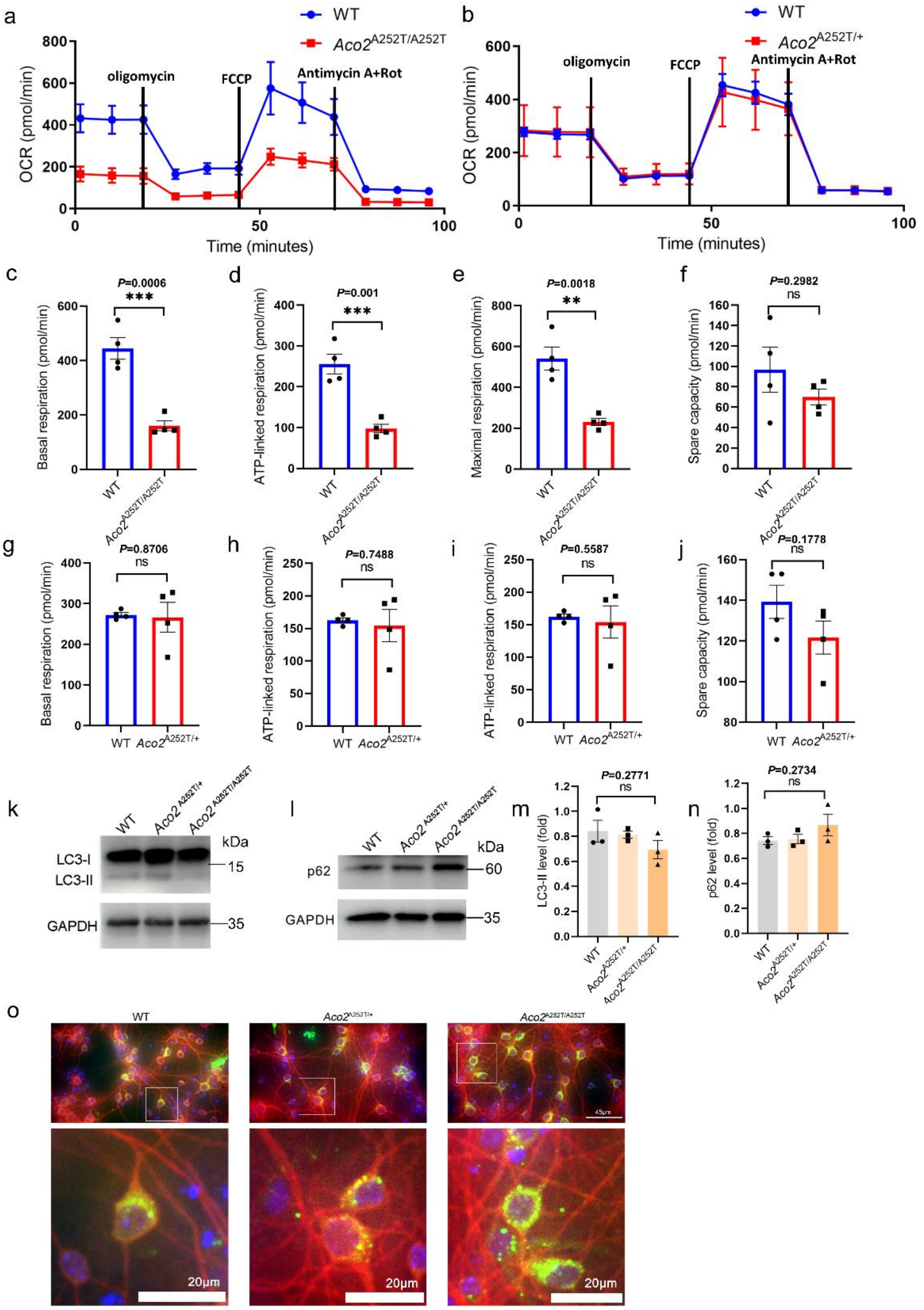
a
WT
Aco2A252T/A252T
oligomycin
FCCP
Antimycin A+Rot
OCR (pmol/min)
Time (minutes)
b
WT
Aco2A252T/+
oligomycin
FCCP
Antimycin A+Rot
OCR (pmol/min)
Time (minutes)
c
P=0.0006
***
Basal respiration (pmol/min)
WT
Aco2A252T/A252T
d
P=0.001
***
ATP-linked respiration (pmol/min)
e
P=0.0018
**
Maximal respiration (pmol/min)
f
P=0.2982
ns
Spare capacity (pmol/min)
g
P=0.8706
ns
Basal respiration (pmol/min)
WT Aco2A252T/+
h
P=0.7488
ns
ATP-linked respiration (pmol/min)
i
P=0.5587
ns
ATP-linked respiration (pmol/min)
j
P=0.1778
ns
Spare capacity (pmol/min)
k
WT
Aco2A252T/+
Aco2A252T/A252T
kDa
LC3-I
LC3-II
15
GAPDH
35
l
p62
60
GAPDH
35
m
P=0.2771
ns
LC3-II level (fold)
n
P=0.2734
ns
p62 level (fold)
o
WT
Aco2A252T/+
Aco2A252T/A252T
20μm

Supplementary Figure 10. The protein levels of DRP1 and MFN2 in the SN of WT and *Aco2*$^{A252T/A252T}$ mice were not affected.

(a and c) Western blot of DRP1 and MFN2. (b and d) Quantification of DRP1 and MFN2 expression in the SN. n=6. Results are expressed as the mean±SEM. Statistical significance was performed with an unpaired t-test between two groups. All the *P* values were two-sided. Source data are available as a Supplementary Data 1 file.

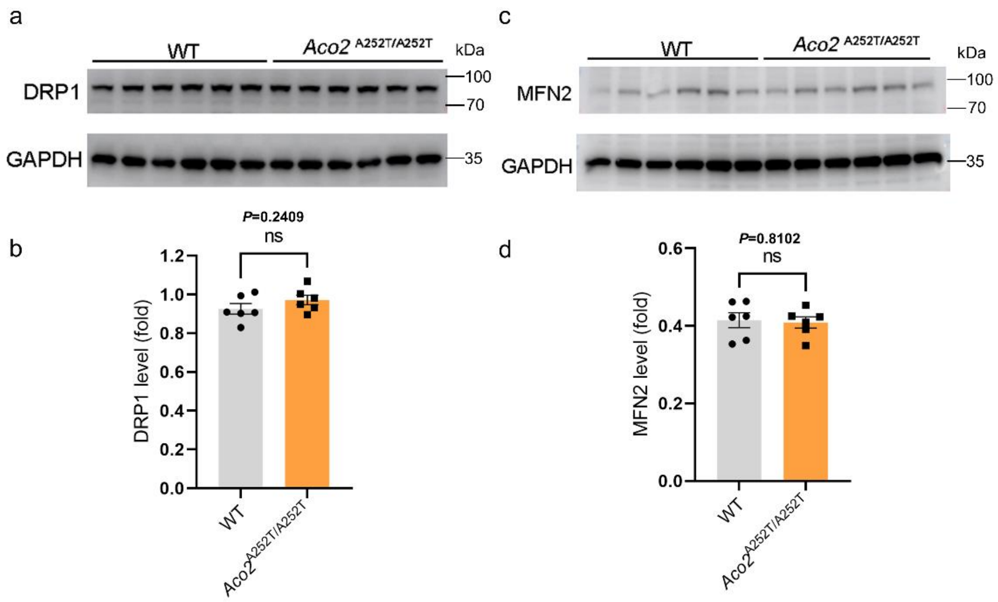

Supplementary Figure 11. The expression of autophagy pathway related proteins was not affected in siRNA-*Aco2*-treated MES23.5 cells.

(a-e) Expression levels of ACO2, p-AMPK, p-ULK1, PI3K III, p-mTOR, p-PI3K, ATG5 and p-Beclin1 in *Aco2*-knock down MES23.5 cells were determined by western blotting.

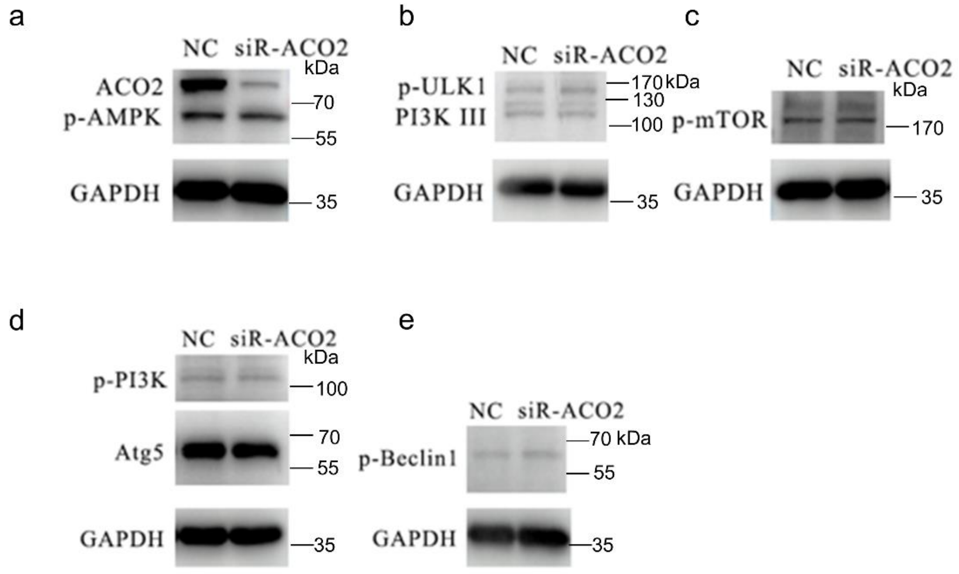

Supplementary Figure 12. Increased PINK1 and Parkin in TA-treated MES23.5 cells and DA neurons of Aco2A252T/A252T mice.

(a and b) Immunofluorescent staining of PINK1 and Parkin (red) in TA-treated MES23.5 cells, the nuclei were stained with DAPI (blue) (scale bars, 10μm). (c and d) Immunofluorescent staining of PINK1 and Parkin (red) in the SN*pc*, TH^+ neurons were stained in green, and the nuclei were stained with DAPI (blue) (scale bars, 10μm, enlarged scale bars, 2μm). n=3. The white arrows: PINK1 and Parkin expressed in TH-positive neurons.

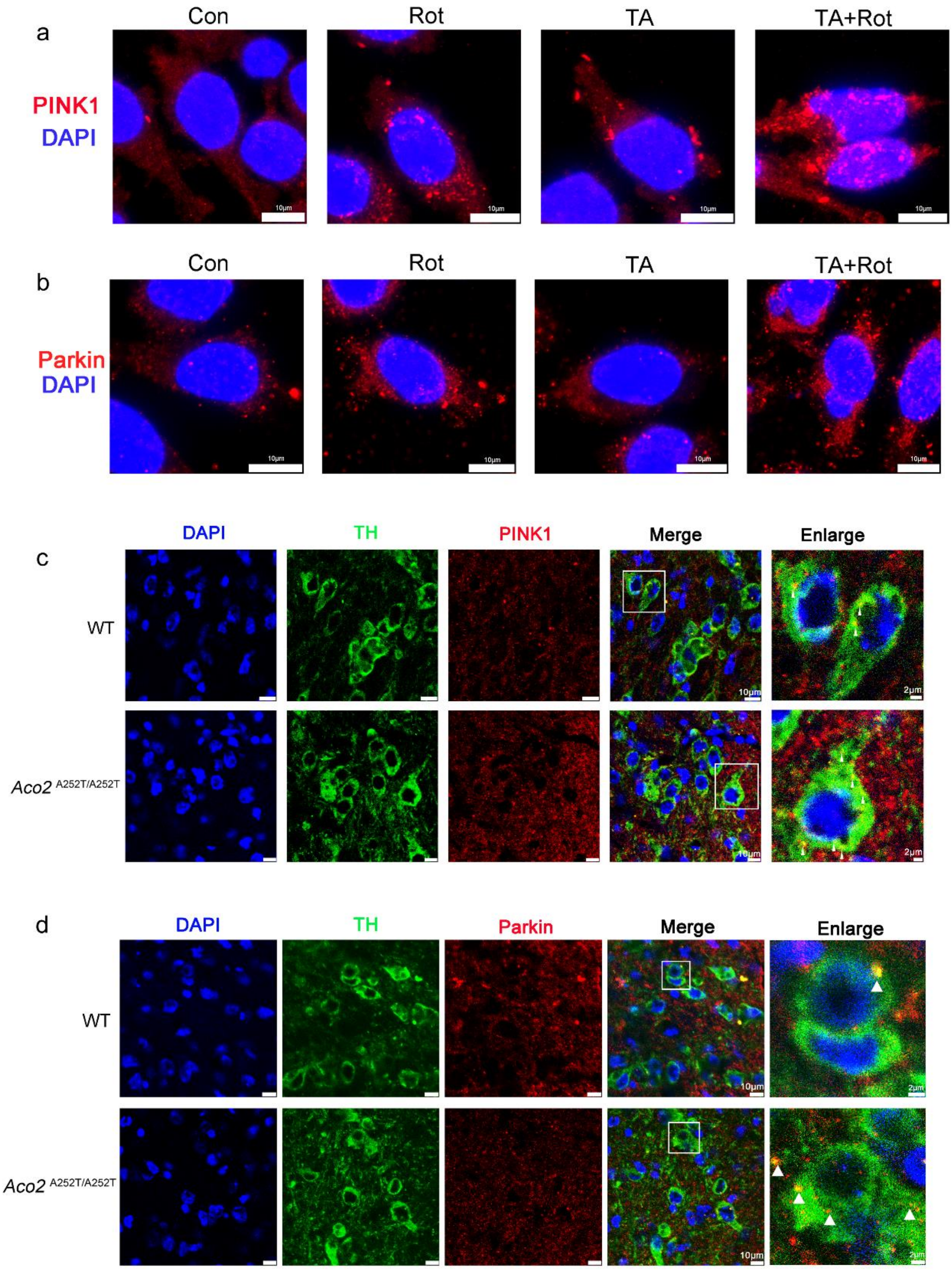
a
Con
Rot
TA
TA+Rot
PINK1
DAPI
10μm
b
Parkin
DAPI
c
DAPI
TH
PINK1
Merge
Enlarge
WT
Aco2 A252T/A252T
2μm
d
Parkin

Supplementary Figure 13. Histone acetylation was increased in PBMCs of PD patients.

(a) Western blot of H3K9ac and H4K5ac. Since H3K9ac and H4K5ac have the same molecular weight as H3 and H4, respectively, we performed experiments on two SDS-PAGE gels separately and provide the results of two membranes. (b and c) Quantification of H3K9ac and H4K5ac in PBMC. n=5 (HC), n=8 (PD). Results are expressed as the mean±SEM. Statistical significance was performed with an unpaired t-test between two groups. All the *P* values were two-sided. Source data are available as a Supplementary Data 1 file.

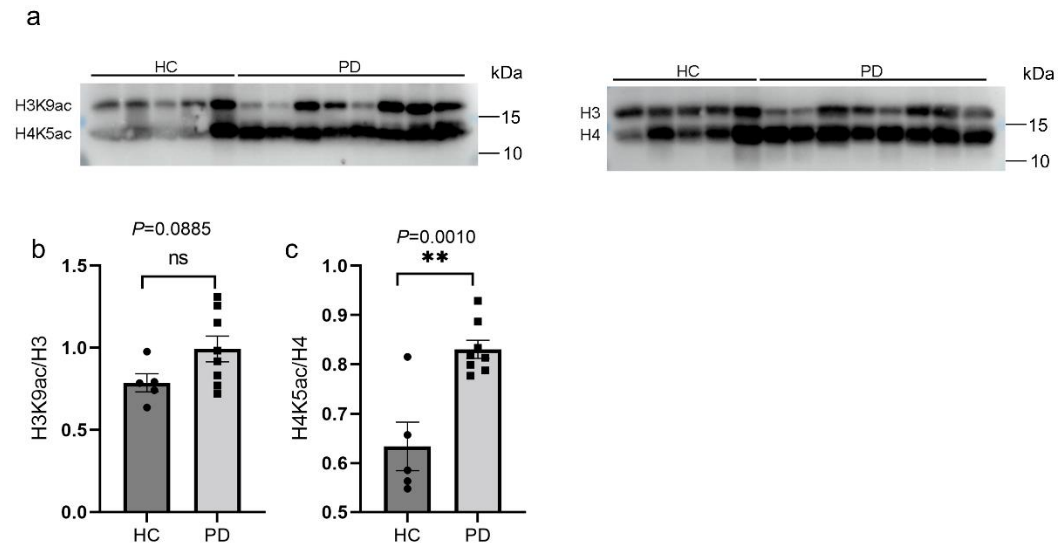

Supplementary Figure 14. ACO2 is not localized in the nucleus of MES23.5 cells. Immunofluorescent staining of ACO2 (green) in MES23.5 cells treated with Rot, TA or siR-*Aco2*, the nuclei were stained with DAPI (blue) (scale bars, 25µm).

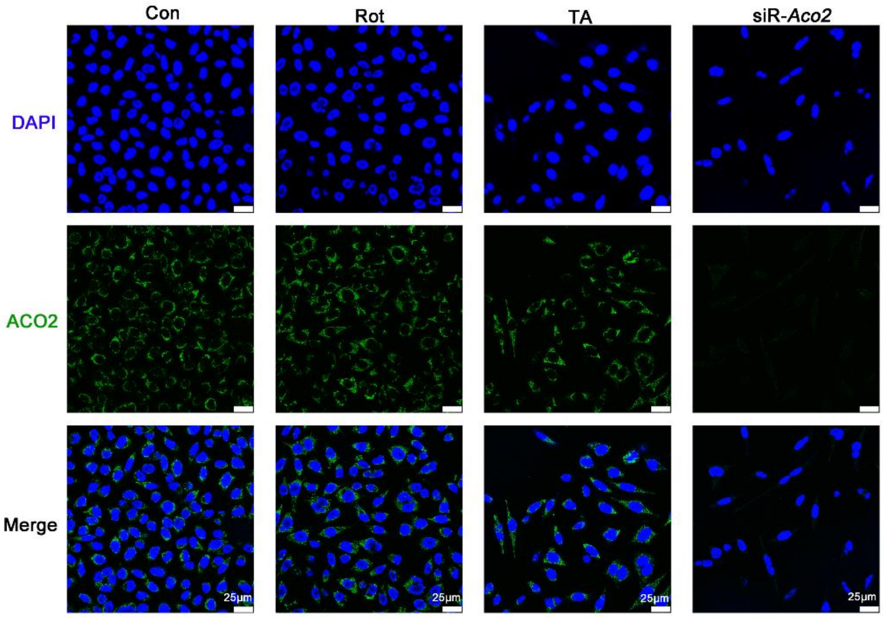

Supplementary Figure 15. Images of uncropped Western blots of Figure 2.

Figure 2g

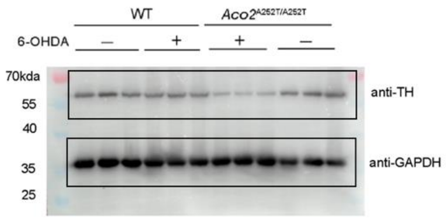

Figure 2h

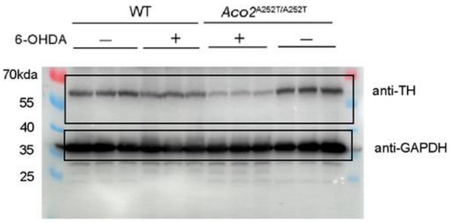

Figure 2l

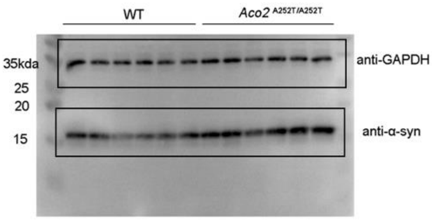

Figure 2m

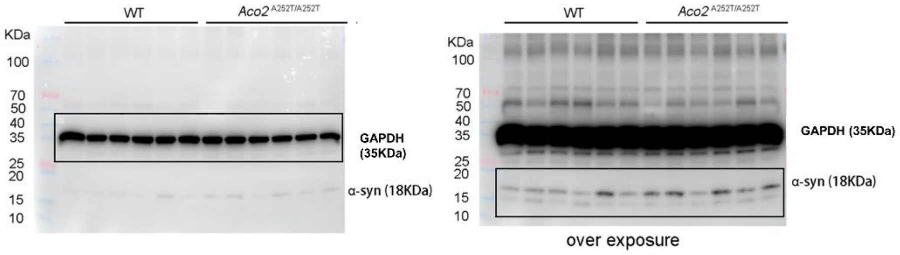

Supplementary Figure 16. Images of uncropped Western blots of Figure 3.

Figure 3k

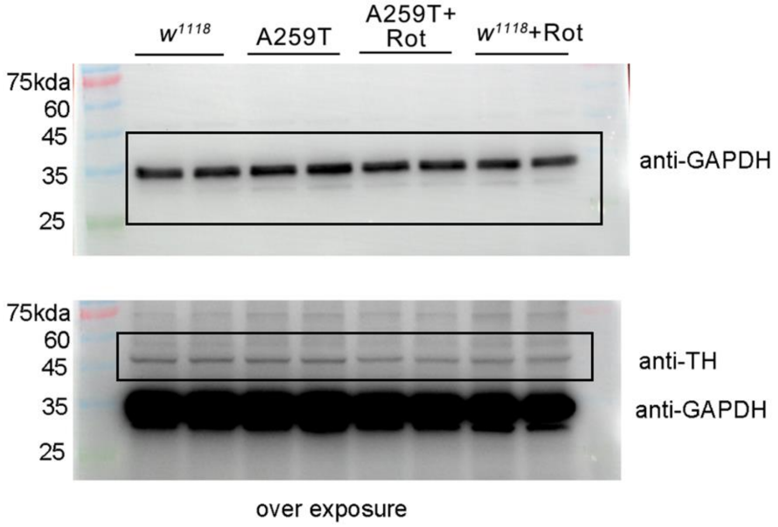

Supplementary Figure 17. Images of uncropped Western blots of Figure 4.

Figure 4c

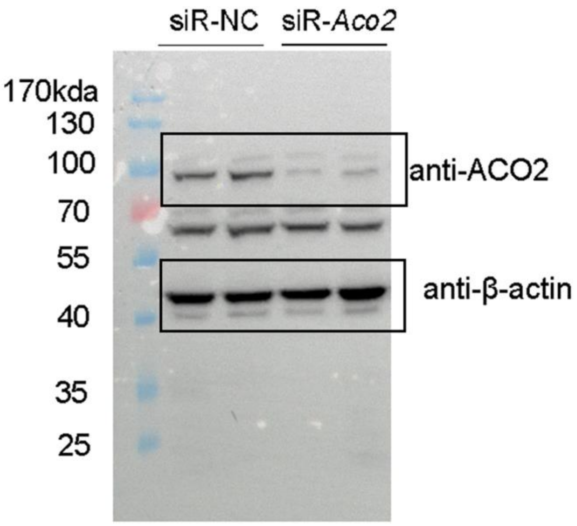

Supplementary Figure 18. Images of uncropped Western blots of Figure 5.

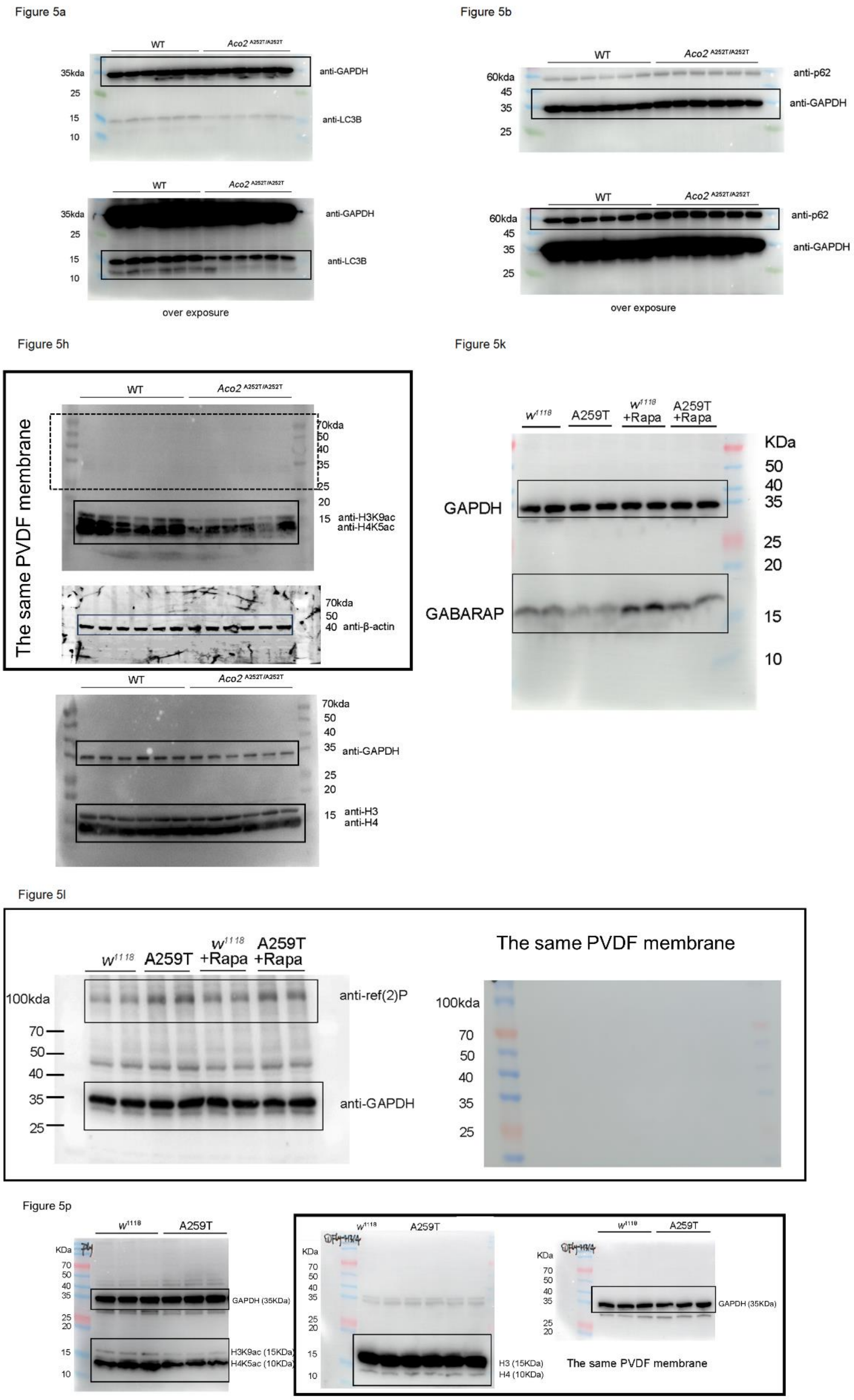

Supplementary Figure 19. Images of uncropped Western blots of Figure 6.

Figure 6a

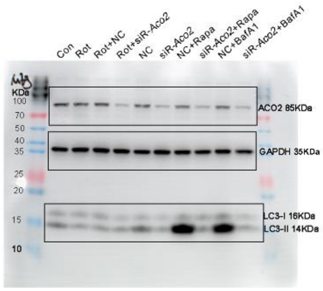

Figure 6b

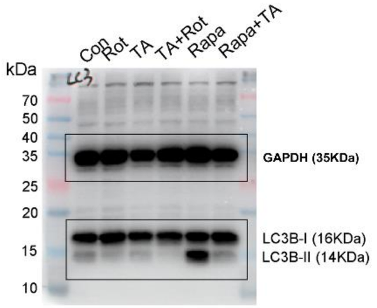

Figure 6c

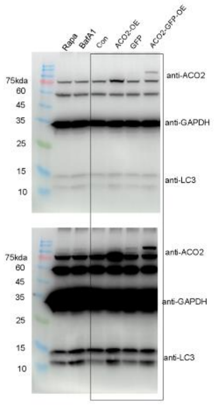

Figure 6g

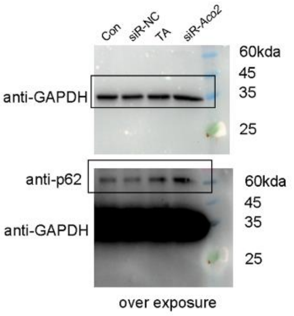

Figure 6l

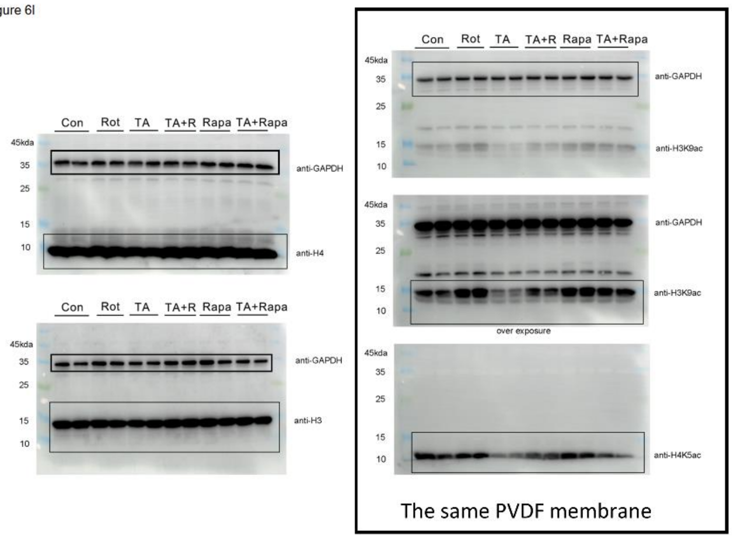

Supplementary Figure 20. Images of uncropped Western blots of Figure 7.

Figure 7c

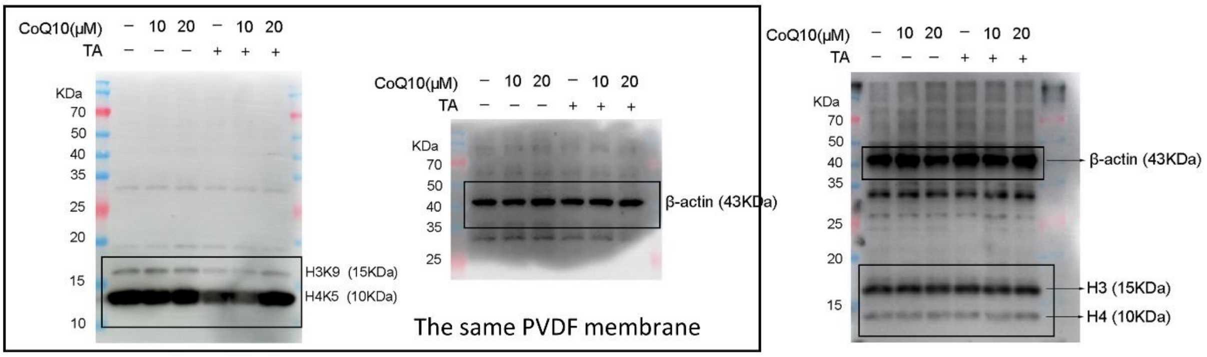

Figure 7d

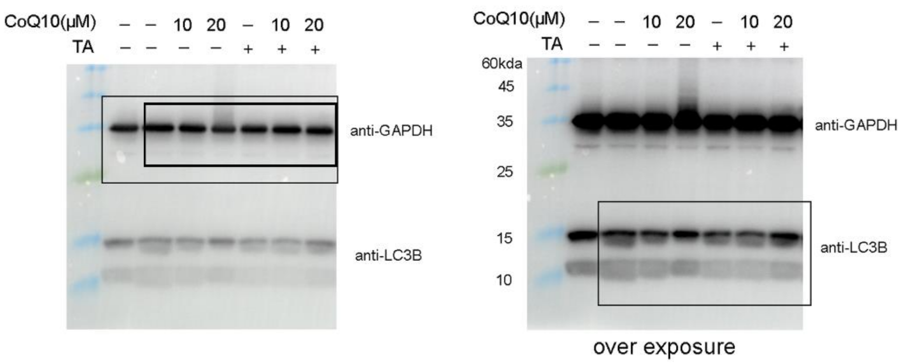

Figure 7k

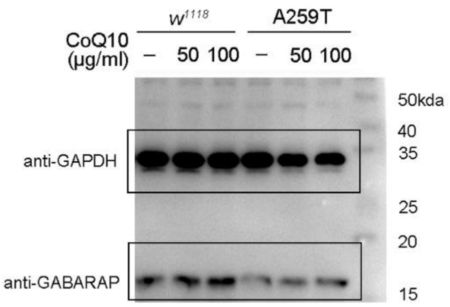

Figure 7l

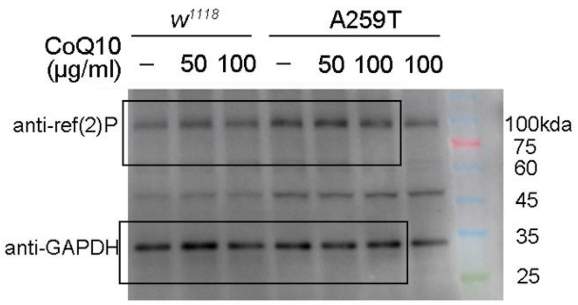

Supplementary Figure 21. Images of uncropped Western blots of Supplementary Figure 3.

Figure S3a

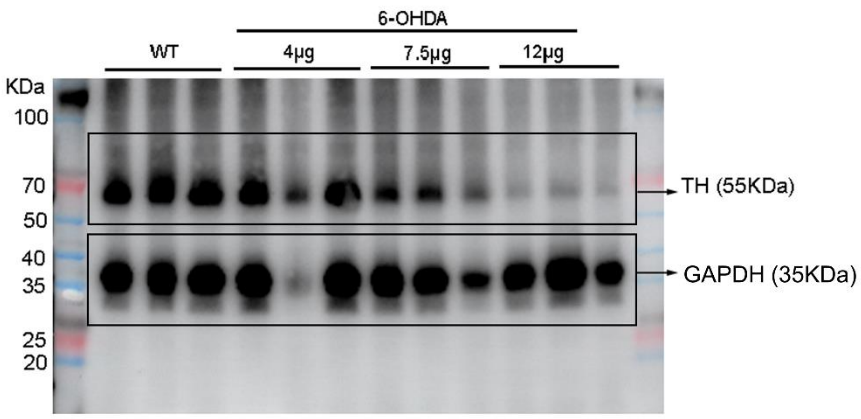

Supplementary Figure 22. Images of uncropped Western blots of Supplementary Figure 5.

Figure S5a

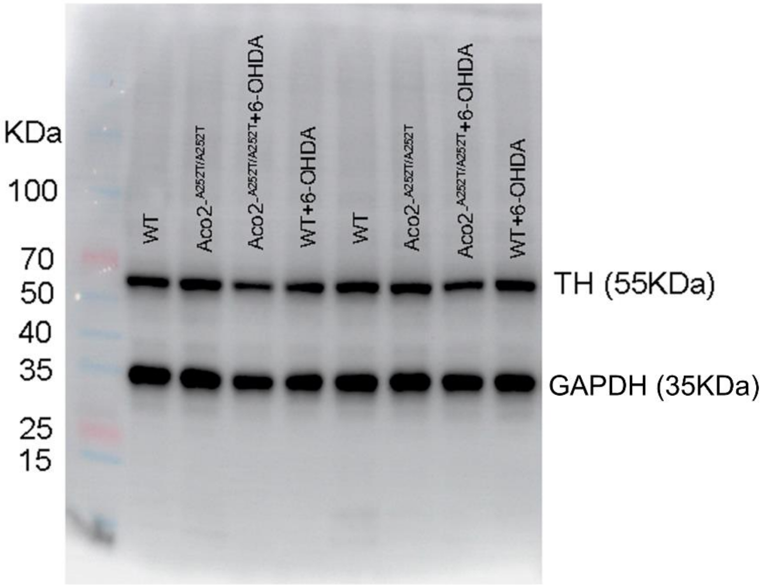

Supplementary Figure 23. Images of uncropped Western blots of Supplementary Figure 6.

Figure S6a

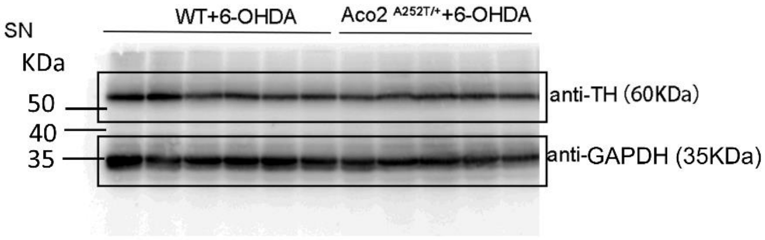

Figure S6b

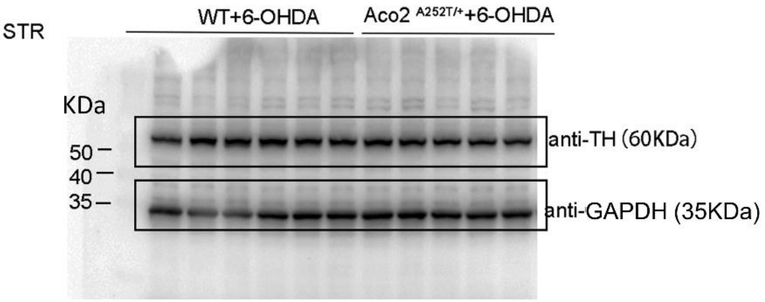

Supplementary Figure 24. Images of uncropped Western blots of Supplementary Figure 9.

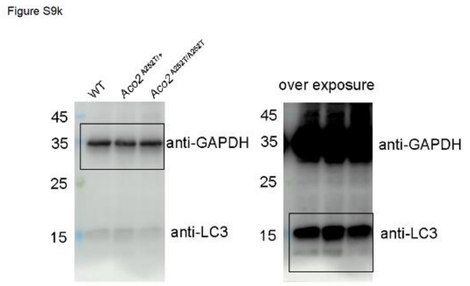

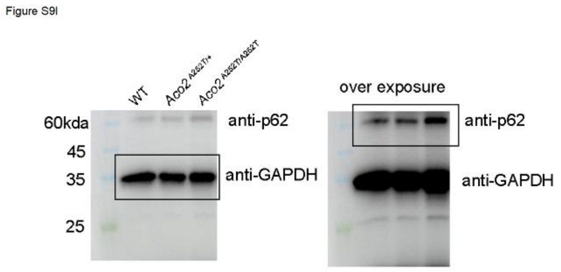

Supplementary Figure 25. Images of uncropped Western blots of Supplementary Figure 10.

Figure S10a

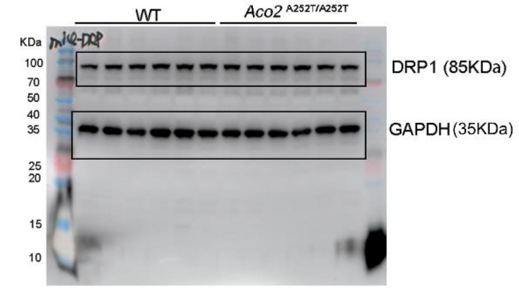

Figure S10c

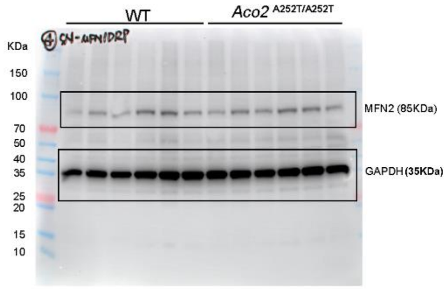

Supplementary Figure 26. Images of uncropped Western blots of Supplementary Figure 11.

Figure S11a

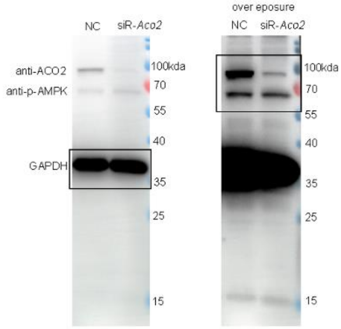

Figure S11b

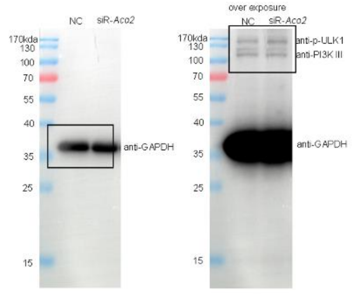

Figure S11c

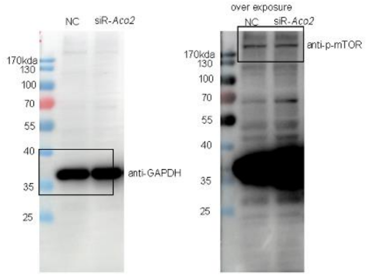

Figure S11d

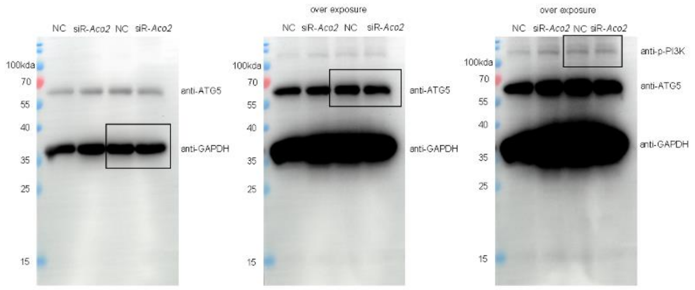

Figure S11e

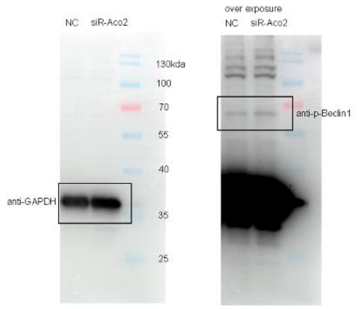

Supplementary Figure 27. Images of uncropped Western blots of Supplementary Figure 13.

Figure S13a

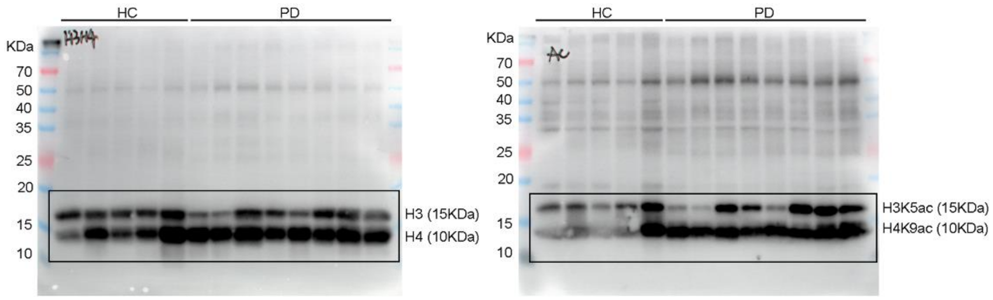

Supplement: Supplementary file 1 — Supplementary information [file 42003_2023_5570_MOESM1_ESM.pdf]
