# Supplementary material for: ACO2 deficiency increases vulnerability to Parkinson’s disease via dysregulating mitochondrial function and histone acetylation-mediated transcription of autophagy genes
Source: Commun Biol. 2023 Nov 25;6:1201. doi: 10.1038/s42003-023-05570-y (PMC10676364; doi:10.1038/s42003-023-05570-y)
Supplement: Supplementary file 4 — Reporting Summary [file 42003_2023_5570_MOESM4_ESM.pdf]

nature portfolio

Corresponding author(s): Chaodong Wang
Last updated by author(s): Nov 9, 2023

Reporting Summary

Nature Portfolio wishes to improve the reproducibility of the work that we publish. This form provides structure for consistency and transparency in reporting. For further information on Nature Portfolio policies, see our Editorial Policies and the Editorial Policy Checklist.

Statistics

For all statistical analyses, confirm that the following items are present in the figure legend, table legend, main text, or Methods section.

n/a	Confirmed	
☐	☒	The exact sample size (*n*) for each experimental group/condition, given as a discrete number and unit of measurement
☐	☒	A statement on whether measurements were taken from distinct samples or whether the same sample was measured repeatedly
☐	☒	The statistical test(s) used AND whether they are one- or two-sided<br>*Only common tests should be described solely by name; describe more complex techniques in the Methods section.*
☒	☐	A description of all covariates tested
☒	☐	A description of any assumptions or corrections, such as tests of normality and adjustment for multiple comparisons
☐	☒	A full description of the statistical parameters including central tendency (e.g. means) or other basic estimates (e.g. regression coefficient) AND variation (e.g. standard deviation) or associated estimates of uncertainty (e.g. confidence intervals)
☐	☒	For null hypothesis testing, the test statistic (e.g. *F*, *t*, *r*) with confidence intervals, effect sizes, degrees of freedom and *P* value noted<br>*Give P values as exact values whenever suitable.*
☒	☐	For Bayesian analysis, information on the choice of priors and Markov chain Monte Carlo settings
☒	☐	For hierarchical and complex designs, identification of the appropriate level for tests and full reporting of outcomes
☒	☐	Estimates of effect sizes (e.g. Cohen's *d*, Pearson's *r*), indicating how they were calculated

Our web collection on statistics for biologists contains articles on many of the points above.

Software and code

Policy information about availability of computer code

Data collection	This study did not generate new codes. The following commercial software were used for data collection:<br>Open field test: lime light 5.0 system (Actimetrics software, USA).<br>Immunofluorescence staining, JC-1 staining: SP8 confocal microscope (Leica Microsystems, Germany).<br>Western blot: chemiluminescence system (Vilber Fusion FX7, French).<br>Mitochondrial stress: Seahorse XFe analyzer (Seahorse Bioscience, USA).<br>Drosophila Activity Monitoring (DAM) system.<br>qRT-PCR: Realtime PCR system (Thermo Fisher, USA).
Data analysis	This study did not generate new codes. The following commercial software were used for data analysis:<br>Statistical tests were performed using GraphPad Prism 9.0 (GraphPad Software, CA) or SPSS via two-tailed unpaired t-test between two groups and one-way ANOVA with Tukey's post hoc test or Dunnett's test for multiple comparisons. Correlations of clinical variables with ACO2 activity of PBMCs were examined by simple linear regression models.<br>Phylogenetic and molecular evolutionary analyses were conducted using MEGA version 11.<br>Analyses of western blot were performed using the ImageJ software.

For manuscripts utilizing custom algorithms or software that are central to the research but not yet described in published literature, software must be made available to editors and reviewers. We strongly encourage code deposition in a community repository (e.g. GitHub). See the Nature Portfolio guidelines for submitting code & software for further information.

Data

Policy information about availability of data

All manuscripts must include a data availability statement. This statement should provide the following information, where applicable:

- Accession codes, unique identifiers, or web links for publicly available datasets
- A description of any restrictions on data availability
- For clinical datasets or third party data, please ensure that the statement adheres to our policy

All materials and data are available upon request. All relevant data generated for this study are available within the Article, Supplementary Information (Supplementary Figure 15-27) and Supplementary Data 1 file. Source data are provided in the Supplementary Information (Supplementary Figure 15-27) and Supplementary Data 1 file.
The raw sequence data reported in this paper have been deposited in the Genome Sequence Archive (Genomics, Proteomics & Bioinformatics 2021) in National Genomics Data Center (Nucleic Acids Res 2022), China National Center for Bioinformation / Beijing Institute of Genomics, Chinese Academy of Sciences (GSA-Human: HRA005774, HRA005403 and HRA005554) that are publicly accessible at https://ngdc.cncb.ac.cn/gsa-human.
All other data are available from the corresponding author (or other sources, as applicable) on reasonable request.

Human research participants

Policy information about studies involving human research participants and Sex and Gender in Research.

Reporting on sex and gender	To evaluate the changes of ACO2 in PD patients, we assayed its expression and enzymatic activity in PBMCs from 114 PD patients (gender: male/female=56/58) and 117 HCs (gender: male/female=51/66).
Population characteristics	1. To evaluate the changes of ACO2 in PD patients, we assayed its expression and enzymatic activity in PBMCs from 114 PD patients (age: 66.02±0.73; gender: male/female=56/58) and 117 HCs (age: 64.12±0.47; gender: male/female=51/66).<br>2. 1,474 PD and 1,456 HCs were recruited and genomic DNA samples were extracted from peripheral blood samples. ACO2 variations were screened by whole-genome sequencing (WGS, 536 HC/460 PD), whole-exome sequencing (WES, 765 HC/771 PD) and panel sequencing (155 HC/243 PD), which were performed for several other research projects.
Recruitment	PD patients and healthy controls (HCs) were recruited during July 2019 and December 2021 from Xuanwu Hospital of Capital Medical University and Community Health Centres of Xinjiekou and Qinlong in Xicheng and Fangshan districts, respectively. The diagnosis of PD was made by at least 2 specialists in PD and movement disorders based on the MDS clinical diagnostic criteria for Parkinson's disease. All the subjects underwent a detailed questionnaire survey and face-to-face PD assessment. The questionnaires provided basic information including sex, current age, Unified Parkinson's Disease Rating Scale (UPDRS), and epidemiological investigation.
Ethics oversight	The study was approved by the ethics committee of Xuanwu Hospital of Capital Medical University.

Note that full information on the approval of the study protocol must also be provided in the manuscript.

Field-specific reporting

Please select the one below that is the best fit for your research. If you are not sure, read the appropriate sections before making your selection.

☒ Life sciences ☐ Behavioural & social sciences ☐ Ecological, evolutionary & environmental sciences

For a reference copy of the document with all sections, see nature.com/documents/nr-reporting-summary-flat.pdf

Life sciences study design

All studies must disclose on these points even when the disclosure is negative.

Sample size	No statistical method was used to predetermine sample size. Sample size was determined in accordance with standard practices in the field.
Data exclusions	No data were excluded from the analysis.
Replication	Reproducibility of experimental findings was assured by repeating at least three times independent experiments, as explained in the text.
Randomization	Samples, mice and Drosophila were randomly allocated to experimental groups.
Blinding	During data analysis, data groups were referred to as numbers, and the grouping status is unknown to analysts.

Reporting for specific materials, systems and methods

We require information from authors about some types of materials, experimental systems and methods used in many studies. Here, indicate whether each material, system or method listed is relevant to your study. If you are not sure if a list item applies to your research, read the appropriate section before selecting a response.

Materials & experimental systems

n/a	Involved in the study
☐	☒ Antibodies
☐	☒ Eukaryotic cell lines
☒	☐ Palaeontology and archaeology
☐	☒ Animals and other organisms
☒	☐ Clinical data
☒	☐ Dual use research of concern

Methods

n/a	Involved in the study
☒	☐ ChIP-seq
☒	☐ Flow cytometry
☒	☐ MRI-based neuroimaging

Antibodies

Antibodies used

1. Western blot:
anti-TH antibody (1:1000, Millipore, ab152), anti-α-synuclein antibody (1:1000, CST, 51510), anti-LC3B antibody (1:1000, proteintech, 14600-1-AP), anti-p62 antibody (1:2000, Abcam, ab56416), anti-H3K9 antibody (1:5000, Active motif, 39137), anti-H4K5 antibody (1:5000, Millipore, 07-327), anti-H3 antibody (1:1000, CST, 9715), anti-H4 antibody (1:1000, CST, 2935), anti-GABARAP antibody (1:1000, Abcam, ab109364), anti-ref(2)P antibody (1:500, Abcam, ab178440), anti-p-AMPK antibody (1:1000, Abcam, ab133448), anti-p-ULK1 antibody (1:1000, CST, 8054), anti-PI3K III antibody (1:1000, CST, 4263), anti-p-mTOR antibody (1:1000, CST, 5536), anti-p-PI3K antibody (1:1000, CST, 17366), anti-ATG5 antibody (1:1000, Abcam, ab108327), anti-p-Beclin1 antibody (1:1000, CST, 35955), anti-DRP1 antibody (1:2000, proteintech, 12957-1-AP), anti-MFN2 antibody (1:1000, Abcam, ab56889), anti-β-actin antibody (1:5000, proteintech, 66009-1-1g), anti-GAPDH antibody (1:5000, proteintech, 60004-1-1g), peroxidase-conjugated anti-mouse or anti-rabbit IgG (1:5000, Zhongshan Golden Bridge Biotechnology, ZB-2305, ZB-2301).
2. Immunofluorescence staining:
anti-TH antibody (1:500, Millipore, ab152), anti-α-synuclein antibody (1:1000, CST, 51510), anti-p62 antibody (1:500, Abcam, ab56416), anti-ref(2)P antibody (1:100, Abcam, ab178440), anti-MAP2 antibody (1:500, Abcam, ab32454).anti-p-α-synuclein antibody (1:500, Abcam, ab51253), anti-PINK1 antibody (1:200, proteintech, 23274-1-AP), anti-Parkin antibody (1:200, proteintech, 14060-1-AP).

Validation

All antibodies used were validated by the commercial source for the applications used in this study.
anti-TH antibody (1:1000, Millipore, ab152):
https://www.sigmaaldrich.cn/CN/en/product/mm/ab152?utm_campaign=Revitalize%20Antibody%204%20-%20China&utm_medium=cpc&utm_source=baidu&utm_content=mm/ab152&utm_term=millipore%20ab152&bd_vid=8898428384503994362
anti-α-synuclein antibody (1:1000, CST, 51510):
https://www.cellsignal.cn/products/primary-antibodies/a-synuclein-e4u2f-xp-rabbit-mab/51510?site-search-type=Products&N=4294956287&Ntt=51510&fromPage=plp&_requestid=338500
anti-LC3B antibody (1:1000, proteintech, 14600-1-AP):
https://www.ptgcn.com/products/MAP1LC3B-Antibody-14600-1-AP.htm
anti-p62 antibody (1:2000, Abcam, ab56416):
https://www.abcam.cn/products/primary-antibodies/sqstm1--p62-antibody-2c11-bsa-and-azide-free-ab56416.html
anti-H3K9 antibody (1:5000, Active motif, 39137):
https://www.activemotif.com/catalog/details/39917/histone-h3-acetyl-lys9-antibody-pab-2
anti-H4K5 antibody (1:5000, Millipore, 07-327):
https://www.sigmaaldrich.cn/CN/en/search/07-327?focus=products&page=1&perpage=30&sort=relevance&term=07-327&type=product
anti-H3 antibody (1:1000, CST, 9715):
https://www.cellsignal.cn/products/primary-antibodies/histone-h3-antibody/9715?site-search-type=Products&N=4294956287&Ntt=9715&fromPage=plp
anti-H4 antibody (1:1000, CST, 2935):
https://www.cellsignal.cn/products/primary-antibodies/histone-h4-l64c1-mouse-mab/2935?site-search-type=Products&N=4294956287&Ntt=2935&fromPage=plp&_requestid=342015
anti-GABARAP antibody (1:1000, Abcam, ab109364):
https://www.abcam.cn/products/primary-antibodies/gabarapgabarapl1gabarapl2-antibody-epr4805-ab109364.html
anti-ref(2)P antibody (1:500, Abcam, ab178440):
https://www.abcam.cn/products/primary-antibodies/ref2p-antibody-ab178440.html
anti-p-AMPK antibody (1:1000, Abcam, ab133448):
https://www.abcam.cn/products/primary-antibodies/ampk-alpha-1-phospho-t183--ampk-alpha-2-phospho-t172-antibody-epr5683-ab133448.html
anti-p-ULK1 antibody (1:1000, CST, 8054):
https://www.cellsignal.cn/products/primary-antibodies/ulk1-d8h5-rabbit-mab/8054?site-search-type=Products&N=4294956287&Ntt=8054&fromPage=plp&_requestid=342093
anti-PI3K III antibody (1:1000, CST, 4263): https://www.cellsignal.cn/products/primary-antibodies/pi3-kinase-class-iii-d9a5-rabbit-mab/4263?_=1680159495314&Ntt=4263&tahead=true

anti-p-mTOR antibody (1:1000, CST, 5536):
https://www.cellsignal.cn/products/primary-antibodies/phospho-mtor-ser2448-d9c2-xp-rabbit-mab/5536?site-search-type=Products&N=4294956287&Ntt=5536&fromPage=plp&_requestid=342270
anti-p-PI3K antibody (1:1000, CST, 17366):
https://www.cellsignal.cn/products/primary-antibodies/phospho-pi3-kinase-p85-tyr458-p55-tyr199-e3u1h-rabbit-mab/17366?site-search-type=Products&N=4294956287&Ntt=17366&fromPage=plp&_requestid=342318
anti-ATG5 antibody (1:1000, Abcam, ab108327) :
https://www.abcam.cn/products/primary-antibodies/apg5latg5-antibody-epr17552-ab108327.html
anti-p-Beclin1 antibody (1:1000, CST, 35955):
https://www.cellsignal.cn/products/primary-antibodies/phospho-beclin-1-ser30-e1c4x-rabbit-mab/35955?site-search-type=Products&N=4294956287&Ntt=35955&fromPage=plp&_requestid=342360
anti-β-actin antibody (1:5000, proteintech, 66009-1-1g):
https://www.ptgcn.com/products/Pan-Actin-Antibody-66009-1-Ig.htm
anti-GAPDH antibody (1:5000, proteintech, 60004-1-1g):
https://www.ptgcn.com/products/GAPDH-Antibody-60004-1-Ig.htm
peroxidase-conjugated anti-mouse or anti-rabbit IgG (1:5000, Zhongshan Golden Bridge Biotechnology, ZB-2305, ZB-2301):
https://www.chem17.com/st236942/Product_22372588.html, https://www.chem17.com/st236942/product_22372518.html
anti-MAP2 antibody (1:500, Abcam, ab32454):
https://www.abcam.cn/products/primary-antibodies/map2-antibody-neuronal-marker-ab32454.html
anti-DRP1 antibody (1:2000, proteintech):
https://www.ptglab.com/products/DNM1L,DLP1-Antibody-12957-1-AP.htm
anti-MFN2 antibody (1:1000, Abcam)
https://www.abcam.cn/products/primary-antibodies/mitofusin-2-antibody-6a8-ab56889.html
anti-p-α-synuclein antibody (1:500, Abcam)
https://www.abcam.cn/products/primary-antibodies/alpha-synuclein-phospho-s129-antibody-ep1536y-ab51253.html
anti-PINK1 antibody (1:200, proteintech)
https://www.ptglab.com/products/PINK1-Antibody-23274-1-AP.htm
anti-Parkin antibody (1:200, proteintech).
https://www.ptglab.com/products/PARK2-Antibody-14060-1-AP.htm

Eukaryotic cell lines

Policy information about cell lines and Sex and Gender in Research

Cell line source(s)	MES23.5 mouse dopaminergic neuron cell line were purchased from the Fenghui Biotechnology (Hunan Fenghui Biotechnology, China).
Authentication	MES23.5 cells were used without modifications once received from the supplier, therefore, they were not authentication.
Mycoplasma contamination	No mycoplasma contamination detected.
Commonly misidentified lines (See ICLAC register)	No commonly misidentified lines.

Animals and other research organisms

Policy information about studies involving animals; ARRIVE guidelines recommended for reporting animal research, and Sex and Gender in Research

Laboratory animals	Aco2-A252T knock-in mice (Aco2A252T/+ mice) were generated by Beijing Biocytogen company (China). Acon-A259T (AconA259T/cyo) knock-in flies were generated by Qidong Fungene Biotechnology (China).
Wild animals	This study did not involve wild animals.
Reporting on sex	Findings involving in vivo experiments apply only to male mice and flies.
Field-collected samples	The study did not involve samples collected from the field.
Ethics oversight	All experimental procedures were approved by the Committee on Animal Care and Usage of Capital Medical University.

Note that full information on the approval of the study protocol must also be provided in the manuscript.
